# Supplementary material for: Programming mRNA decay to modulate synthetic circuit resource allocation
Source: Nat Commun. 2017 Apr 26;8:15128. doi: 10.1038/ncomms15128 (PMC5414051; doi:10.1038/ncomms15128)
Supplement: Supplementary Information — Supplementary Figures, Supplementary Tables, Supplementary Note and Supplementary References [file ncomms15128-s1.pdf]

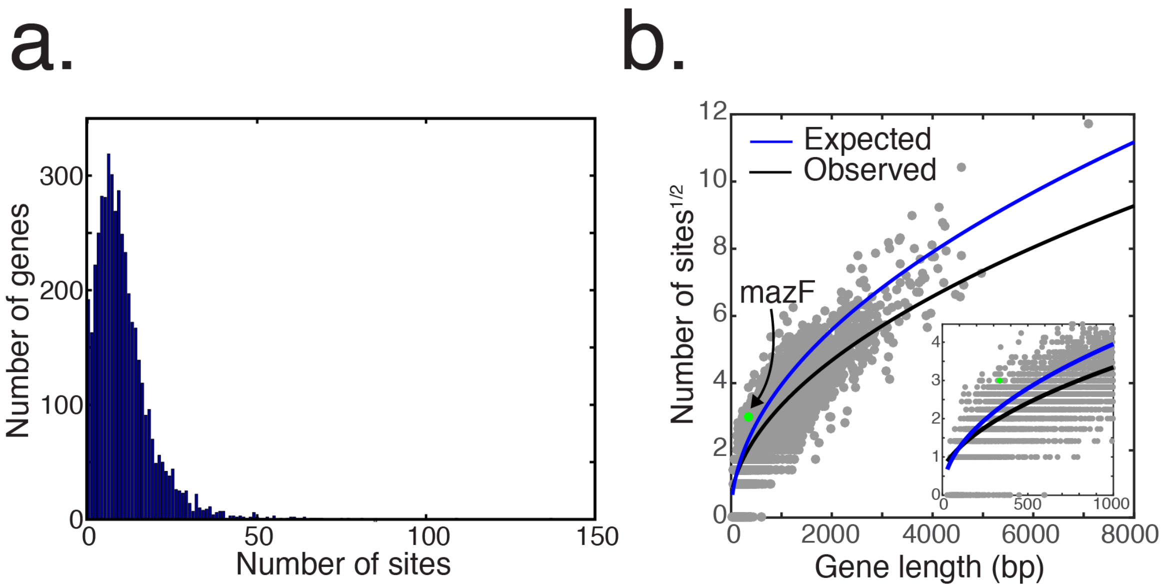

Supplementary Figure 1. MazF recognition sites in *E. coli* coding sequences. **(a)** Distribution of MazF recognition sites in *E. coli* coding sequences. 96% of coding sequences contain at least one recognition site. **(b)** Relationship between gene length and the square root of the number of recognition sites for each transcript. Expected relationship based on the GC content of the *E. coli* genome (blue line) and observed relationship (black line) using robust linear regression. *mazF* is highlighted (green data point) and contains a larger number of MazF sites than predicted. Scatter plot of gene length vs. square root of the number of sites across a limited range of values (inset).

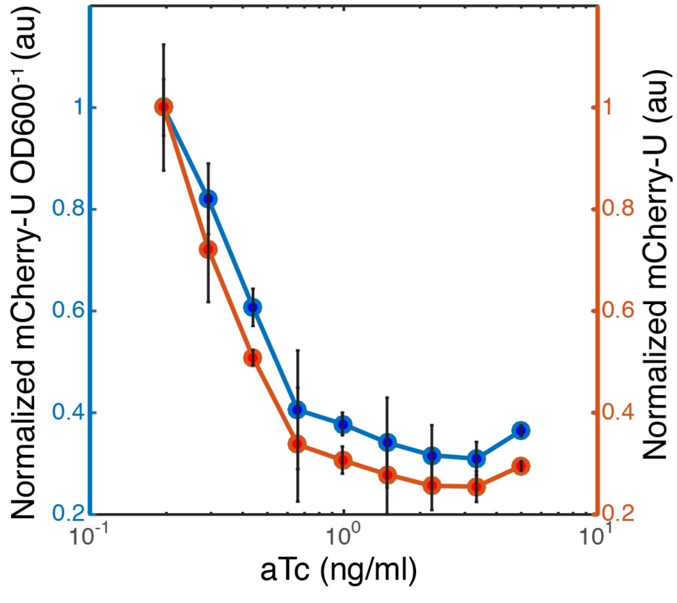

Supplementary Figure 2. Relationship between the concentration of MazF and the expression of an unprotected fluorescent reporter (mCherry-U). *mazF* and *mCherry-U* were regulated by an aTc-inducible promoter (P_{TET}) and arabinose-inducible promoter (P_{BAD}), respectively. Total fluorescence or fluorescence divided by OD600 was normalized to the maximum value across conditions. Cells were induced with 0.05% arabinose for 14.2 hr. Error bars represent 1 s.d. (n = 4).

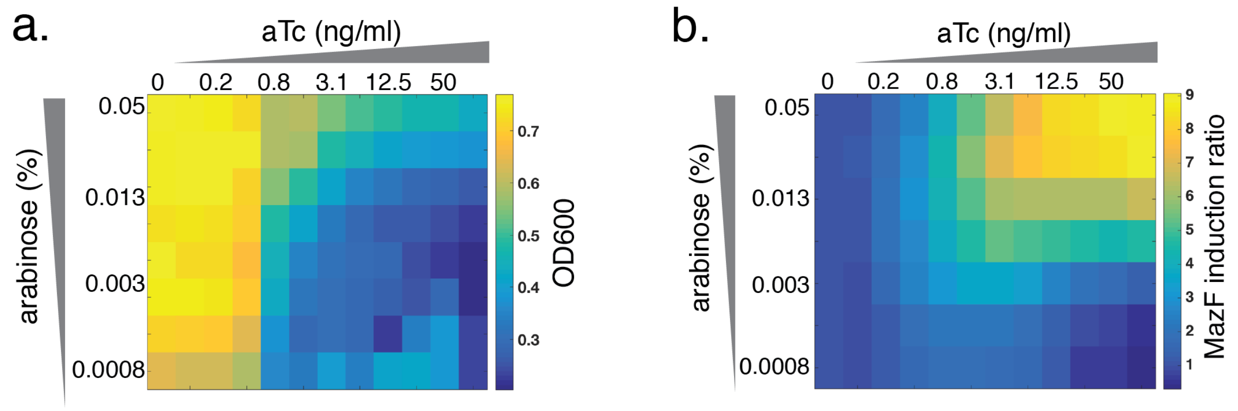

Supplementary Figure 3. MazF activity inhibited growth and enhanced the expression of a protected fluorescent reporter (mCherry-P). MazF and mCherry-X were regulated by an aTc (P_{TET}) and arabinose-inducible (P_{BAD}) promoter, respectively. **(a)** OD600 at saturation across a range of aTc and arabinose concentrations. Cells were induced for 10 hr. **(b)** MazF induction ratio of mCherry-P fluorescence divided by OD600 across a range of arabinose and aTc concentrations. Cells were induced for 10 hr. We measured the expression of mCherry-P divided by OD600 in the presence and absence of MazF induction. The induction ratio is defined as the division of the former quantity by the latter.

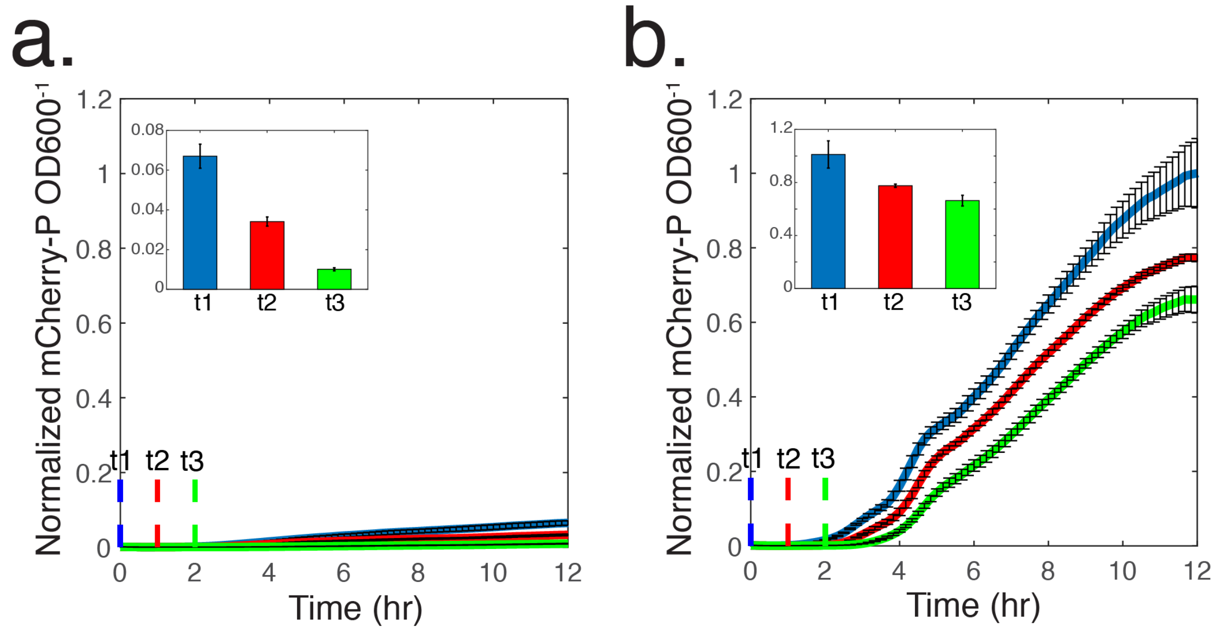

Supplementary Figure 4. Relationship between the timing of induction of mCherry-P and time-resolved measurements of gene expression in the presence (5 ng ml^{-1} aTc) or absence (0 ng ml^{-1} aTc) of MazF. mCherry-P and MazF were controlled by an IPTG (P_{LAC}) and aTc-inducible promoter (P_{TET}). mCherry-P was divided by OD600 and normalized to maximum steady-state expression value across all conditions. **(a)** Normalized mCherry-P expression as a function of time for uninduced cells (0 ng ml^{-1} aTc). 1 mM IPTG was administered to different cell populations at three times highlighted by the dashed lines (t1 = 0 hr, t2 = 1 hr and t3 = 2 hr). Bar plot of normalized mCherry-P expression for the t1, t2 and t3 conditions following 12 hours of induction (inset). **(b)** Normalized mCherry-P expression as a function of time for cells induced with MazF (5 ng ml^{-1} aTc). Cells were induced with IPTG at three times highlighted by the

dashed lines. Bar plot of normalized mCherry-P expression for the t1, t2 and t3 conditions following 12 hr of induction (inset). Error bars represent 1 s.d. (n = 4).

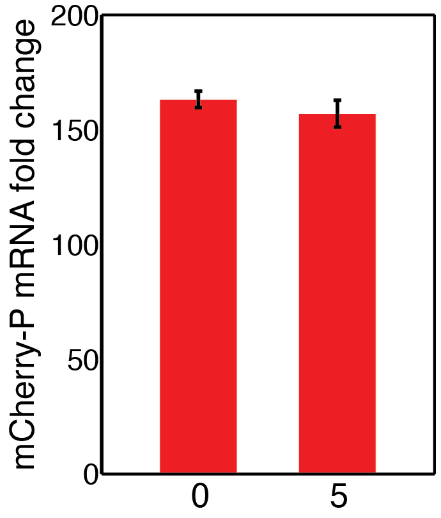

Supplementary Figure 5. *mCherry-P* mRNA fold change measured by qPCR in the absence (0 ng ml^{-1} aTc) or presence (5 ng ml^{-1} aTc) of MazF following 56 min of induction. Cells were induced with 0.05% arabinose. MazF and mCherry-P were controlled by an aTc (P_{TET}) and arabinose-inducible (P_{BAD}) promoter, respectively. Error bars represent 1 s.d. (n = 3).

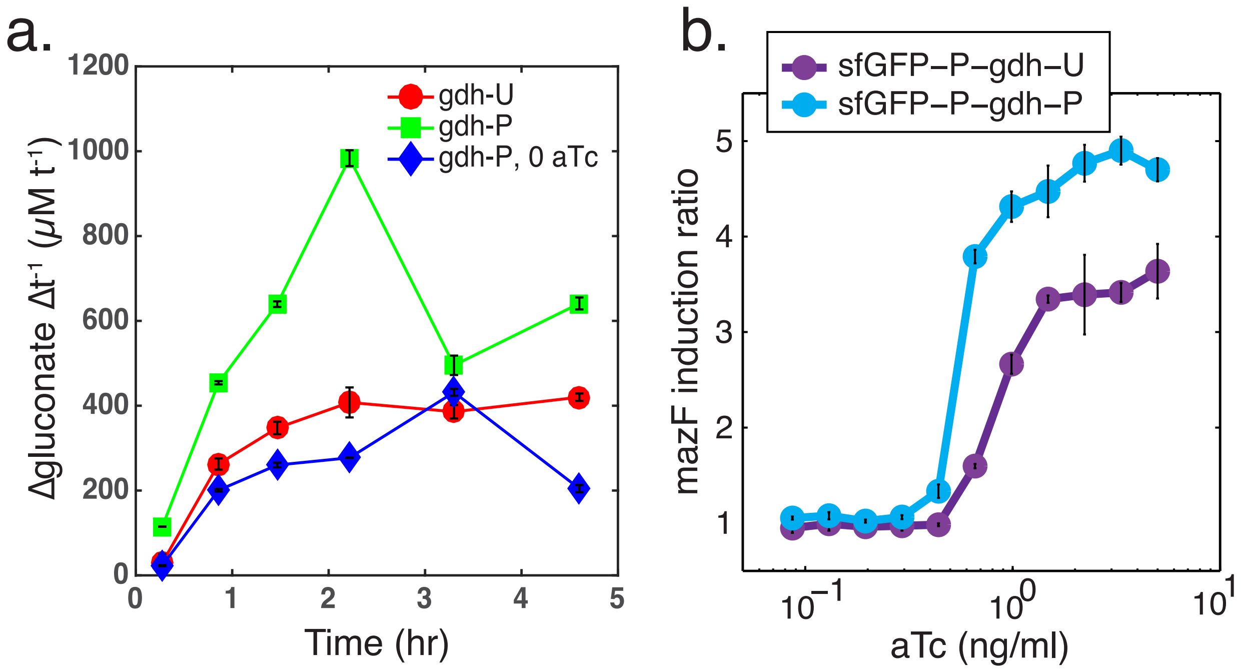

Supplementary Figure 6. MazF resource allocator significantly enhanced gluconate flux and the expression of a protected fluorescent protein fusion (sfGFP-P) to glucose dehydrogenase (Gdh). MazF was regulated by an aTc-inducible promoter (P_{TET}). The protected and unprotected

versions of Gdh and sfGFP-Gdh were controlled by an IPTG-inducible promoter (P_{LAC}). **(a)** Change in gluconate concentration per unit time for cell populations expressing an unprotected version of Gdh (gdh-U) or protected Gdh (gdh-P) driven by an IPTG-inducible promoter (P_{LAC}) in the presence (5 ng ml^{-1} aTc) or absence of MazF (0 ng ml^{-1} aTc). The media was supplemented with 1.5% glucose and 1 mM IPTG. **(b)** MazF induction ratio of an N-terminal fusion of sfGFP-P to Gdh-U or Gdh-P as a function of aTc following 12.2 hr of induction with 1 mM IPTG. The media was supplemented with 1.5% glucose. We measured the expression of sfGFP-P divided by OD600 in the presence and absence of MazF induction. The induction ratio is defined as the division of the former quantity by the latter. Error bars represent 1 s.d. from the mean of three technical replicates (n = 3).

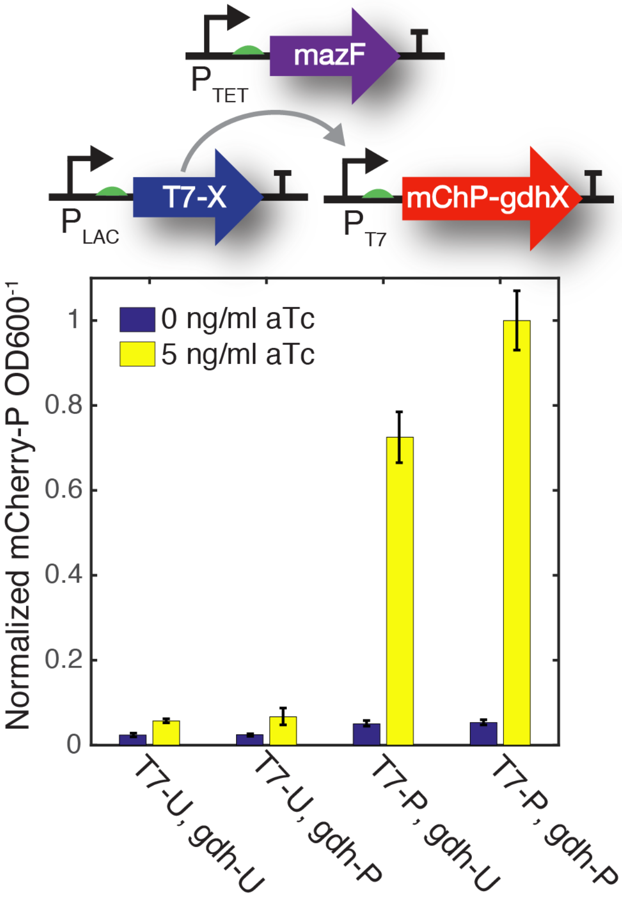

Supplementary Figure 7. Protection of T7 RNA polymerase (T7-P) enhanced the expression of mCherry-P N-terminally fused to Gdh-X (X represents U or P) following MazF induction. Fluorescence divided by OD600 was normalized to the maximum expression across all conditions. Cells were induced with 0 or 5 ng ml^{-1} aTc and 1 mM IPTG for 10.8 hr. The media was supplemented with 1.5% glucose. Error bars represent 1 s.d. (n = 6). Strain S2 (Supplementary Table I) was used for this experiment. T7-X and mCherry-X were controlled by an IPTG (P_{LAC}) and T7 (P_{T7}) regulated promoter, respectively.

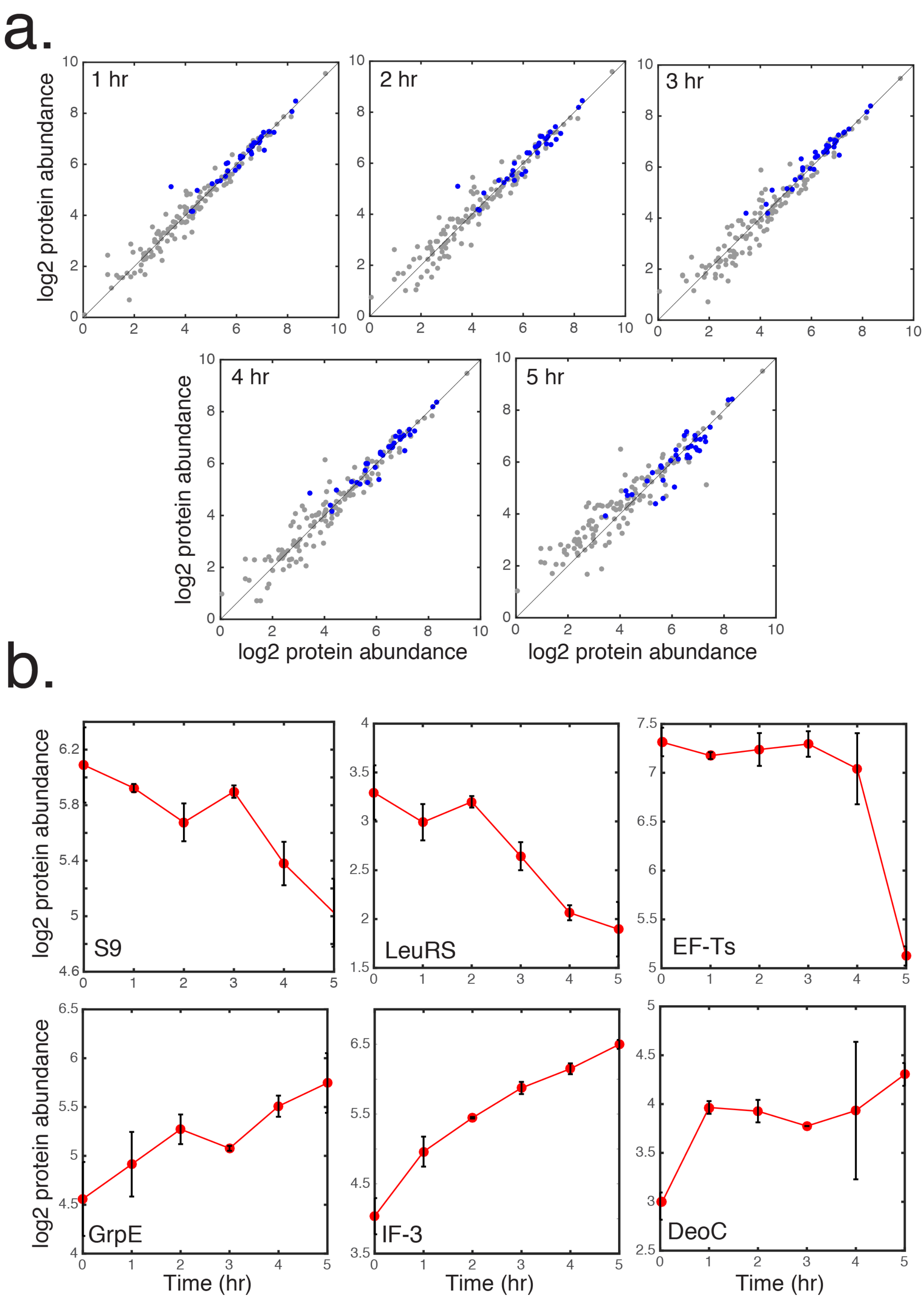
a.
1 hr
2 hr
3 hr
4 hr
5 hr
log2 protein abundance
log2 protein abundance
log2 protein abundance
b.
S9
LeuRS
EF-Ts
GrpE
IF-3
DeoC
log2 protein abundance
Time (hr)

Supplementary Figure 8. Time-series proteomics measurements of cell populations (strain S2 in Supplementary Table I) induced with MazF (5 ng ml^{-1} aTc). **(a)** Scatter plots of log2 transformed protein abundance (normalized total spectra) of 216 proteins prior to induction with MazF (x-axis) vs. exposure to MazF for 1-5 hr (y-axis). Ribosomal proteins are highlighted in blue. **(b)** Representative proteins that significantly decreased (top row) or increased (bottom row) in response to aTc administration (5 ng ml^{-1}). Error bars represent 1 s.d. (n = 4).

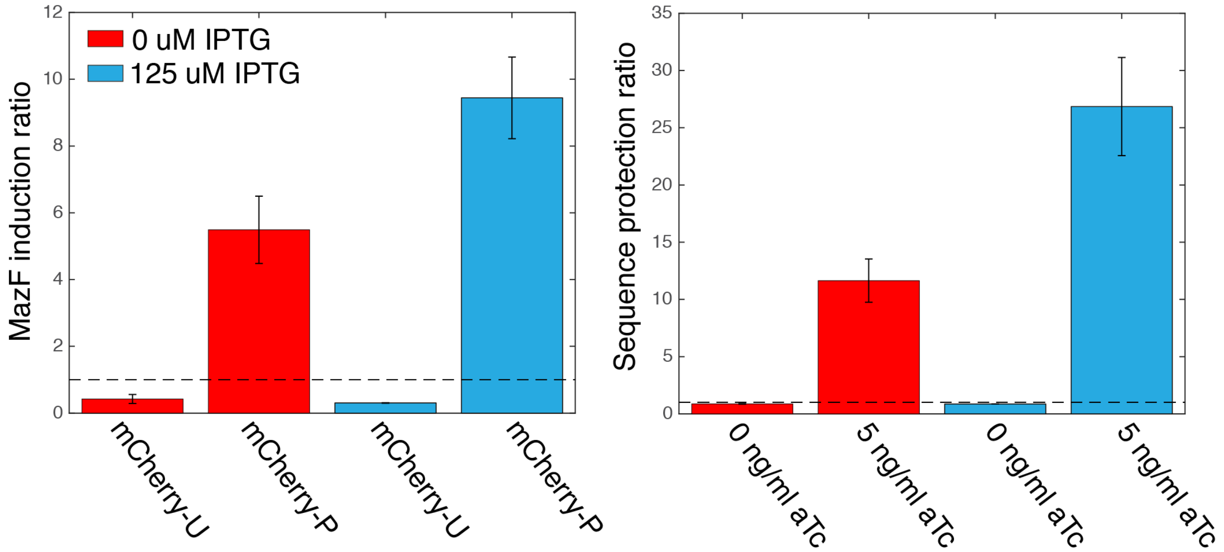

Supplementary Figure 9. Co-expression of support genes protected RNAse R (RNAse R-P) and EF-Ts (EF-Ts-P) did not improve the resource redistribution activity compared to cells induced with a single protected support gene RNAse R-P or EF-Ts-P (Figure 3b). MazF was controlled by an aTc-inducible promoter and RNAse R-P and EF-Ts-P were regulated by distinct IPTG-inducible promoters. We measured the expression of mCherry-P divided by OD600 in the presence and absence of MazF induction. The induction ratio is defined as the division of the former quantity by the latter. The sequence protection ratio is defined as the ratio of mCherry-P $OD600^{-1}$ to mCherry-U $OD600^{-1}$ in the presence or absence of MazF. MazF induction ratio (left) or sequence protection ratio (right) of mCherry-P in cells in the presence or absence of RNAse R-P and EF-Ts-P. Cells were induced with 125 mM IPTG and 0 or 5 ng ml^{-1} aTc for 14.2 hr. Error bars represent 1 s.d. (n = 2).

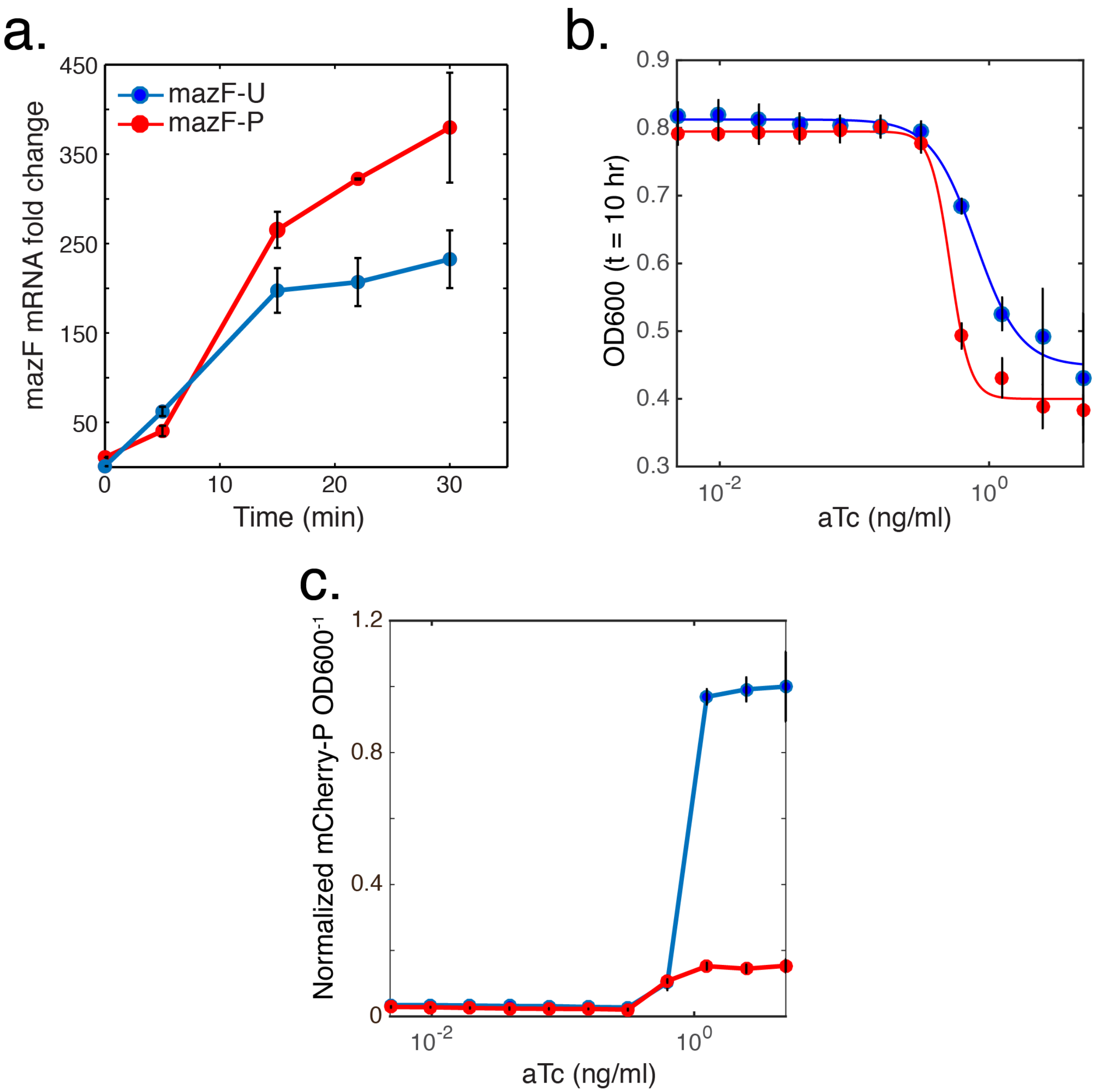

Supplementary Figure 10. Characterization of aTc-inducible MazF-U or MazF-P on *mazF* mRNA dynamics, growth and mCherry-P expression. MazF and mCherry-P were regulated by an aTc (P_{TET}) and arabinose-inducible promoter (P_{BAD}). **(a)** *mazF* mRNA fold change measured by qPCR as a function of time for cells induced with MazF-P or MazF-U. Error bars represent 1 s.d. (n = 2). **(b)** Saturating cell density (OD600) as a function of aTc following 11.2 hr of induction. Error bars represent 1 s.d. (n = 4). **(c)** Normalized expression of mCherry-P as a function of aTc. Cells were induced with 0.05% arabinose and a range of aTc concentrations for 11.2 hr. Strain S3 and plasmids P1-2,4 were used for this experiment. Error bars represent 1 s.d. (n = 4).

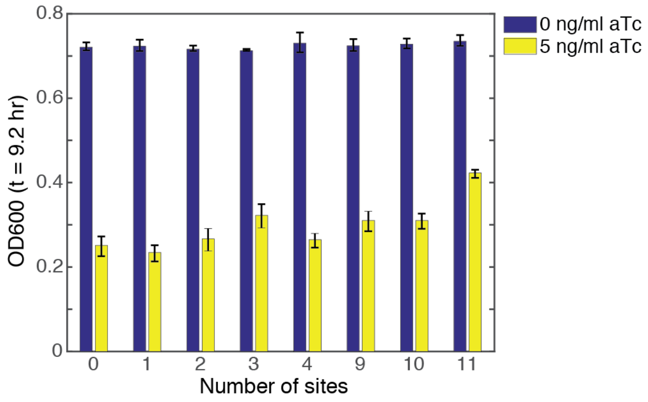

Supplementary Figure 11. Saturating cell densities (OD600) of cell populations in the absence (0 ng ml^{-1} aTc) or presence (5 ng ml^{-1} aTc) of induction with a set of *mazF* variants. MazF was regulated by an aTc-inducible promoter (P_{TET}). The x-axis indicates the number of MazF recognition sites in the *mazF* mRNA sequence (plasmids P37-43 in Supplementary Table I). Cells were induced with 0.05% arabinose for 9.2 hr. Error bars represent 1 s.d. (n = 4).

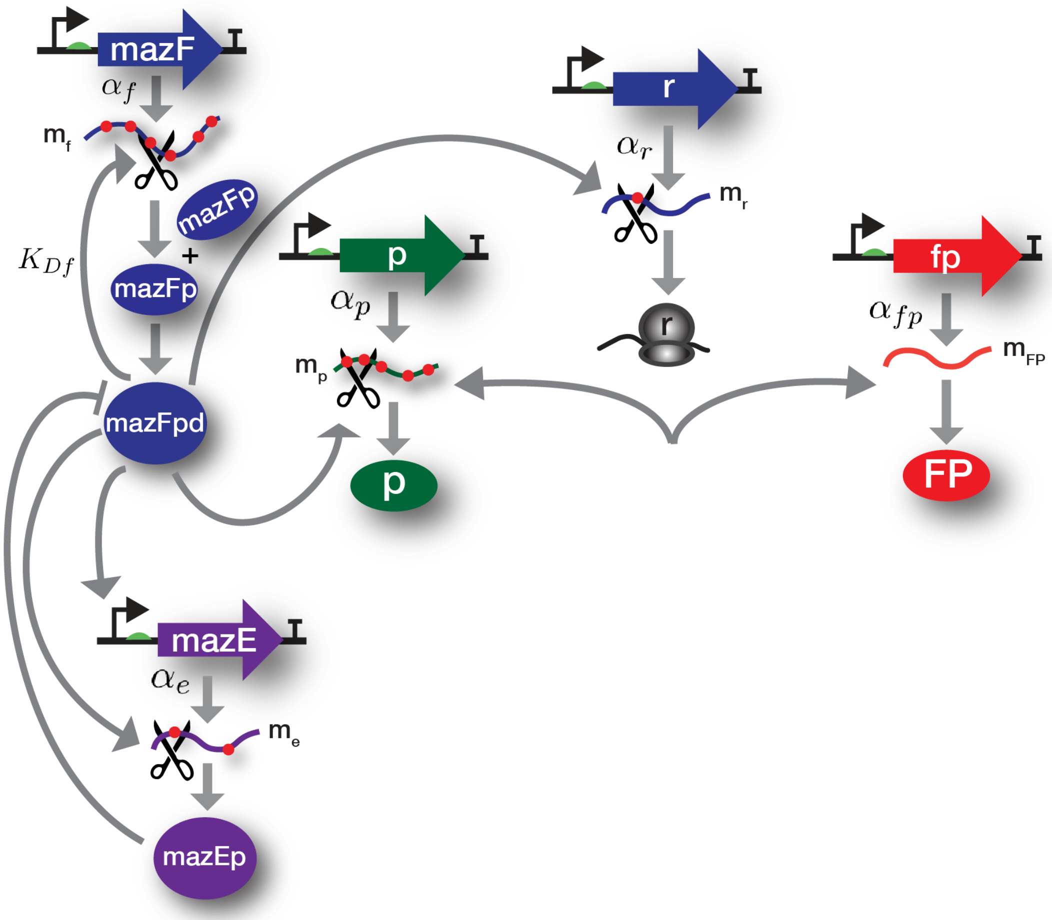

Supplementary Figure 12. Schematic of the MazF resource allocator circuit. MazF monomer (*mazFp*) forms a dimer (*mazFpd*) that targets the transcripts encoding the proteome *p*, ribosome *r*, *mazF* and *mazE* for decay referred to as m_p, m_r, m_f and m_e, respectively. m_f does not contain MazF recognition sizes and is thus protected from MazF-induced mRNA decay. *mazFpd*

induces the synthesis of MazE protein (*mazEp*), which inhibits *mazFpd* activity via molecular sequestration. The transcription rates of m_f, m_p, m_e, m_{fp} and m_r are represented as α_f, α_p, α_e, α_{fp} and α_r, respectively. The dissociation constant of *mazFpd* to m_f is denoted as K_{Df}. Protein synthesis of the protected gene *FP* is enhanced by alterations in resource partitioning as a consequence of MazF-dependent mRNA decay.

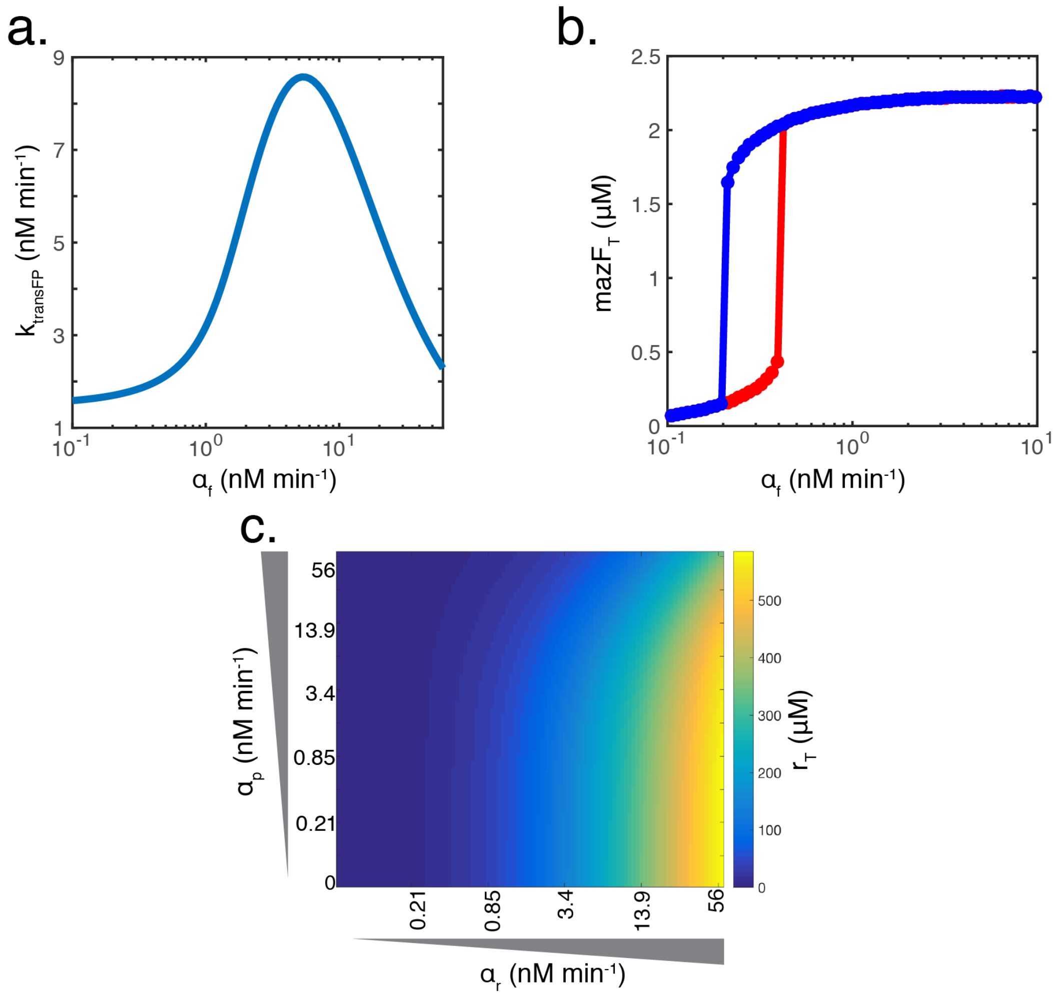

Supplementary Figure 13. Non-monotonic circuit performance, trade-offs and bistability in the dynamic resource allocation model in the absence of MazE (α_e = 0). **(a)** Resource redistribution activity scored by the steady-state (t = 278 hr) protected gene (*FP*) translation rate ($k_{transFP}$) as a function of the *mazF* transcription rate α_f for MazF dissociation constant to the *mazF* transcript K_{Df} = 40 nM. K_{Df} represented the MazF mRNA-decay negative feedback loop strength. **(b)** $mazF_T$ as a function α_f for K_{Df} = 3.86 μM, corresponding to weak negative feedback, for two initial conditions (blue and red correspond to high or low initial concentrations of *r* and *mazFp*). Here, $mazF_T = 2^*[pf]_{ss} + 2^*[rf]_{ss} + 2^*[ff]_{ss} + 2^*[fe]_{ss} + 2^*[mazFpd]_{ss} + [mazFp]_{ss}$, where ss denotes steady-state. The model exhibited bistability across a range of α_f values. **(c)** Total ribosome concentration at steady-state (r_T) as a function of the transcription rate of *r* (α_r) and *p* transcription rate (α_p) for α_f, α_{FP} = 0. Model species and parameter values are listed in Supplementary Table II and III.

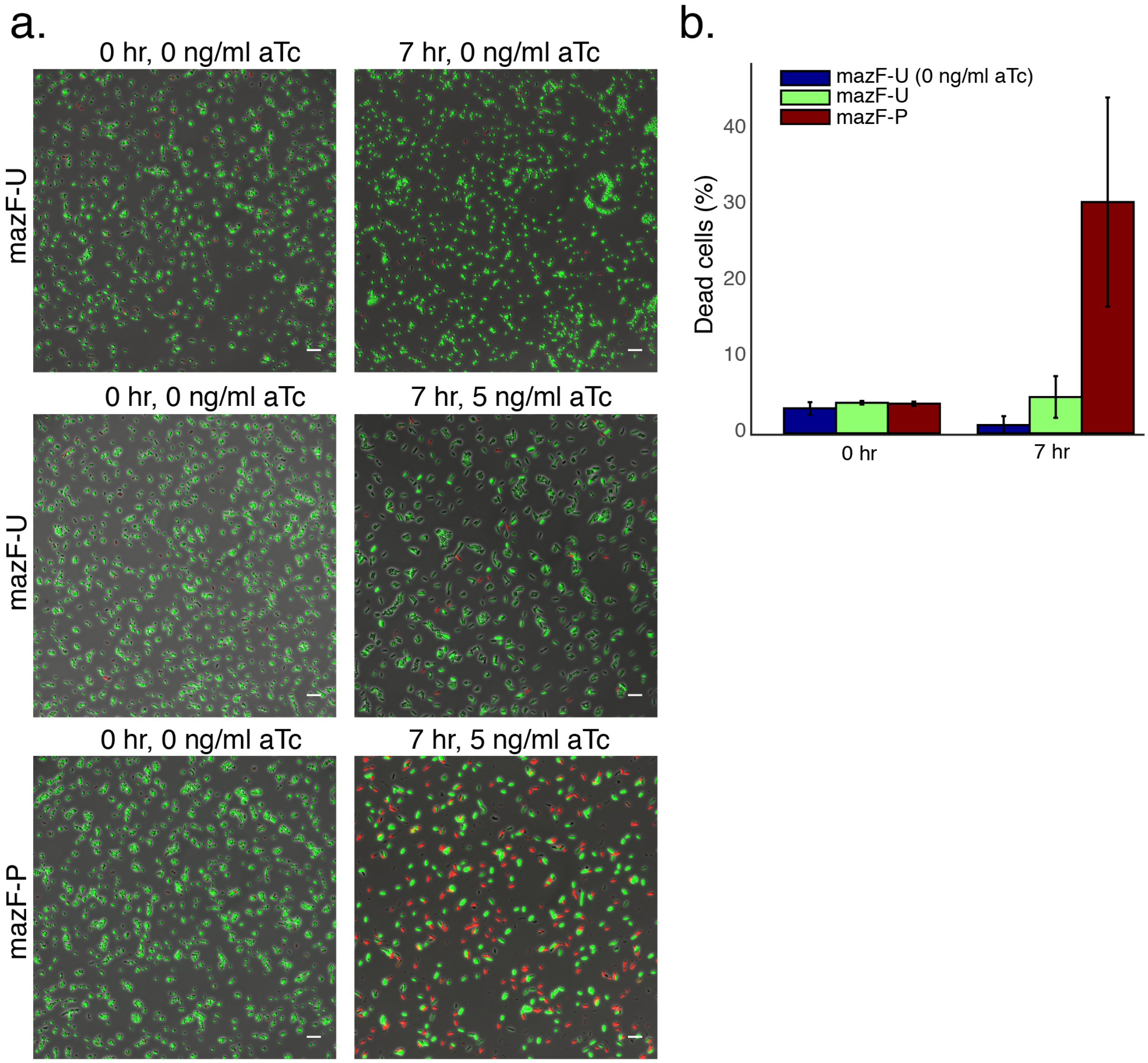

Supplementary Figure 14. Characterization of cell viability using the LIVE/DEAD assay. Strain S3 bearing plasmid P1 or P2 were used in this experiment (Supplementary Table I). **(a)** Representative fluorescence microscopy images of cells uninduced (0 ng ml^{-1} aTc) or induced (5 ng ml^{-1} aTc) with MazF-U or MazF-P for 0 or 7 hr. Red (propidium iodine) and green (SYTO 9) represents cells with compromised and intact membranes, respectively. Scale bars represent 10 µm. **(b)** Ratio of the number of cells with compromised membranes over the total number of cells for each condition following 0 (n = 3502, 3413, 4844 cells from left to right) or 7 hr of induction (n = 2455, 1706, 1653 cells from left to right). Error bars represent 1 s.d. (n = 2).

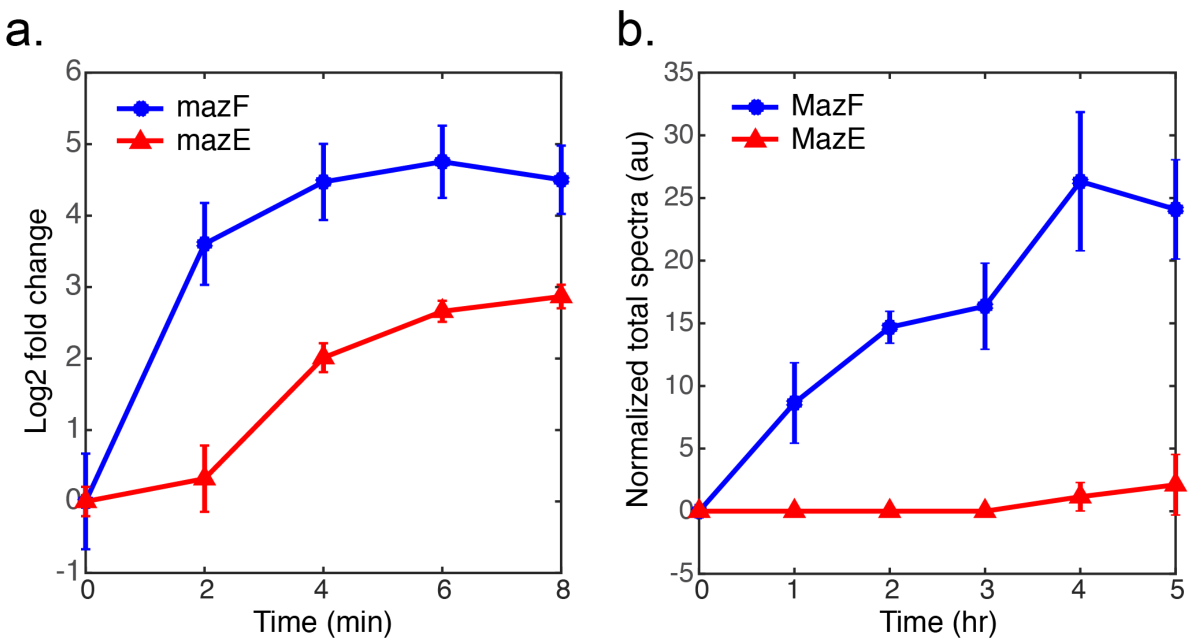

Supplementary Figure 15. Temporal variation in the transcript and protein levels of MazE and MazF in cells (strain S2 in Supplementary Table I) induced with 5 ng ml^{-1} aTc. MazF is regulated by an aTc-inducible promoter (P_{TET}). **(a)** RNA-seq log2 fold change of *mazE* and *mazF* transcripts as a function of time. Error bars represent 1 s.d. (n = 2). **(b)** Protein abundance (normalized total spectra) of MazE and MazF as a function of time in proteomics data. Error bars represent 1 s.d. (n = 4).

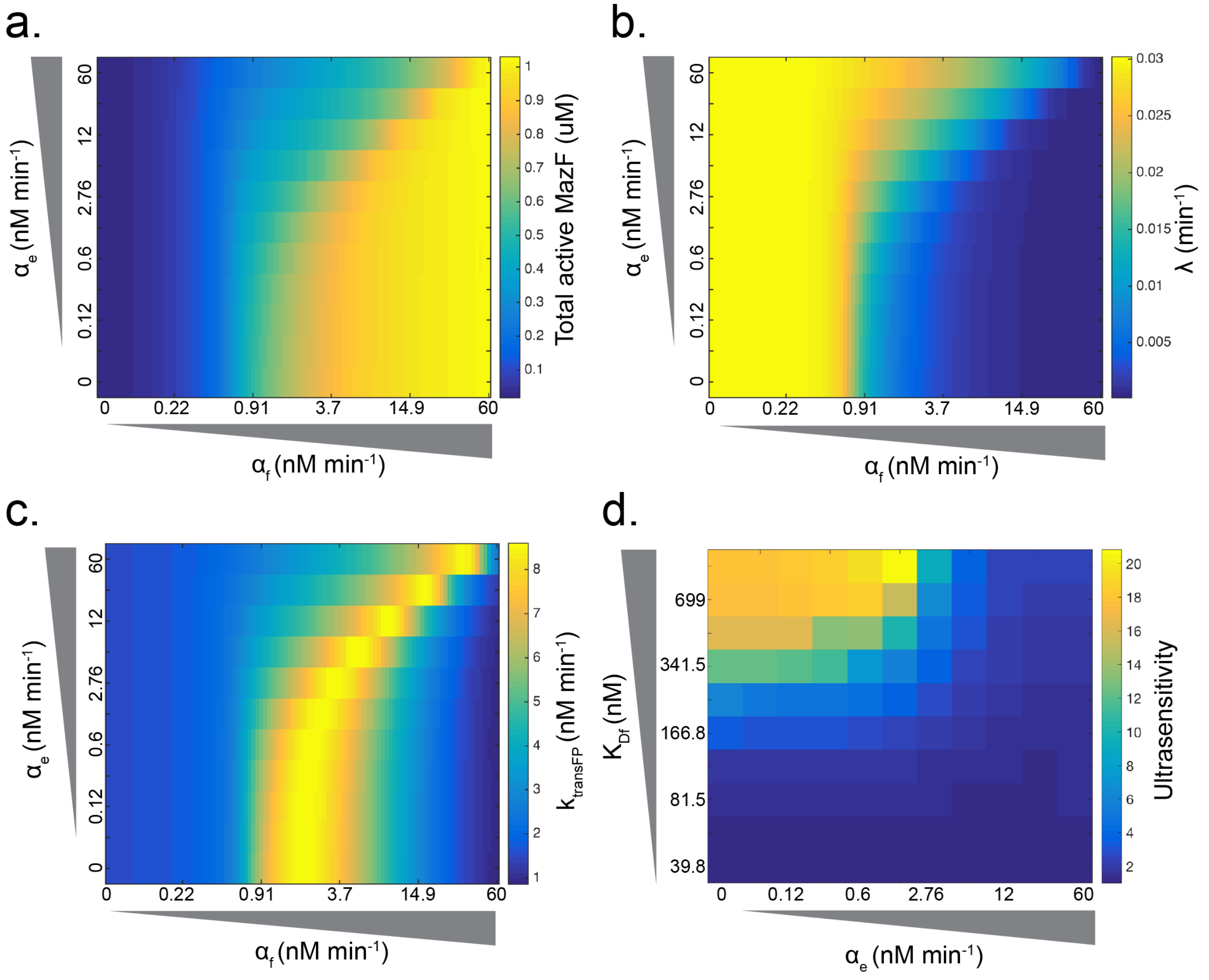

Supplementary Figure 16. The MazE negative feedback loop influences circuit properties and growth. **(a)** Steady-state total active MazF (total active MazF is equal to $[pf]_{ss}$ + $[rf]_{ss}$ + $[ff]_{ss}$ + $[fe]_{ss}$ + $[mazFpd]_{ss}$, where ss denotes the steady-state concentration) as a function of the *mazF* and *mazE* transcription rates α_f and α_e. The binding affinity of MazF to the *mazF* transcript (K_{Df}) was equal to 116.6 nM. **(b)** Steady-state growth rate (λ) as a function of α_f and α_e for K_{Df} = 116.6 nM. **(c)** Steady-state translation rate of a protected gene *FP* ($k_{transFP}$) across a range of α_f and α_e values for K_{Df} = 116.6 nM. **(d)** Maximum logarithmic sensitivity (ultrasensitivity) of the dose response of α_f vs. total steady-state MazF ($mazF_T$) concentration across a range of α_e and K_{Df} values. Here, $mazF_T = 2*[pf]_{ss} + 2*[rf]_{ss} + 2*[ff]_{ss} + 2*[fe]_{ss} + 2*[mazFpd]_{ss} + 2*[cef]_{ss} + [mazFp]_{ss}$.

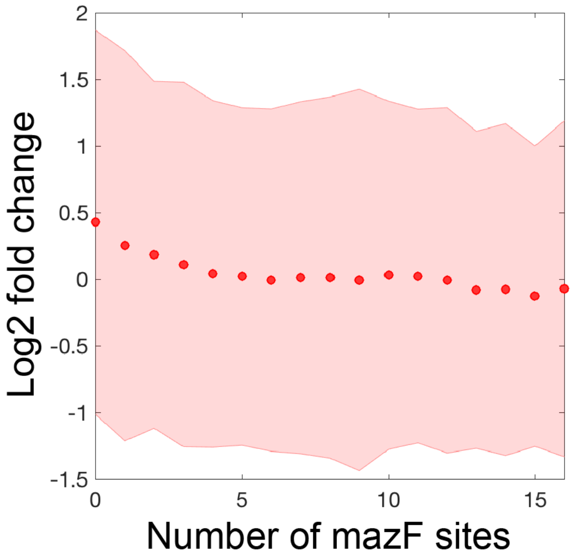

Supplementary Figure 17. Scatter plot of the number of *mazF* sites vs. mean log2 fold change in transcript abundance following administration of 5 ng ml^{-1} aTc for 8 min to strain S2 (Supplementary Table I). A 5-point moving average was applied to the data. The shaded region denotes 1 s.d. from the log2 transformed mean RPKM (n = 2) for all transcripts containing a fixed number of sites.

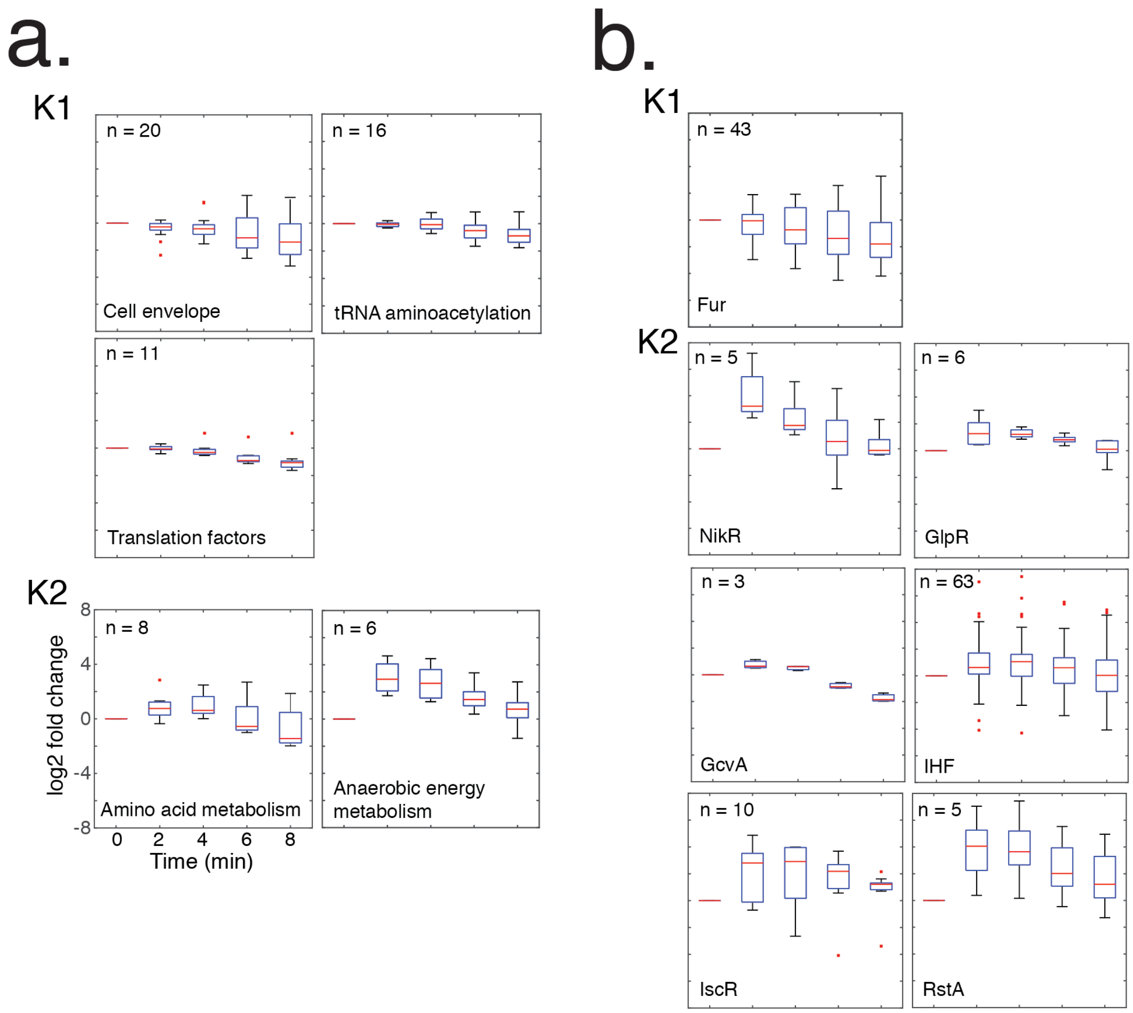

Supplementary Figure 18. Functional (TIGRFAM) and regulatory (RegulonDB) gene enrichment using a Fishers exact test in RNA-seq clusters shown in Figure 5c. **(a)** Box plots showing the RNA-seq log2 fold change (y-axis) as a function of time (x-axis) for each category. On each box, the red line indicates the median, the bottom and top edges represent the 25th and 75th percentiles and '+' denote outlier data points. n denotes the number of genes in each category. **(a)** Functional enrichments in clusters ($p < 0.05$ using the Fisher's exact test). **(b)** Regulatory enrichments in clusters ($p < 0.05$ using the Fisher's exact test). Enriched categories are listed in Supplementary Table V.

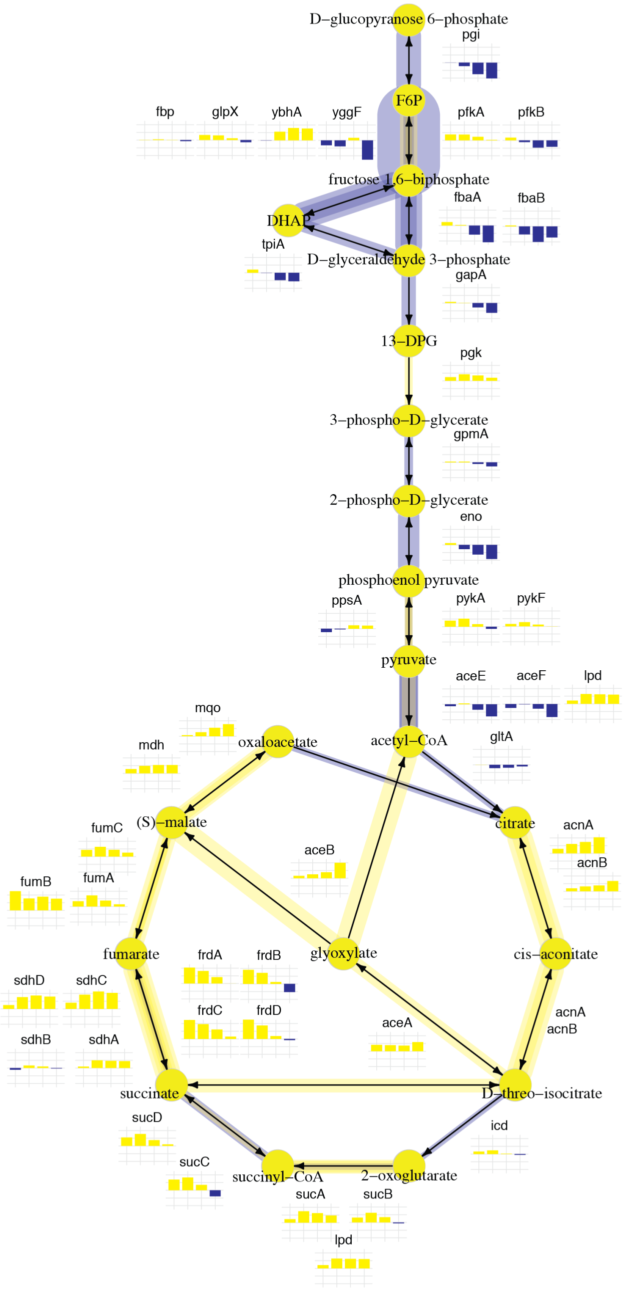

Supplementary Figure 19. Schematic of the glycolysis and TCA cycle metabolic network highlighting the RNA-seq log2 fold-change following 8 min of induction with MazF (5 ng ml-1 aTc). Each node represents a metabolite and the directed edges denote enzymes that catalyze the reactions. Yellow and blue denote an increase or decrease in transcript abundance following aTc administration. The width of the edges represents the magnitude of the log2 fold change. The time-series log2 fold-change of transcript abundance for each gene is represented by a bar graph.

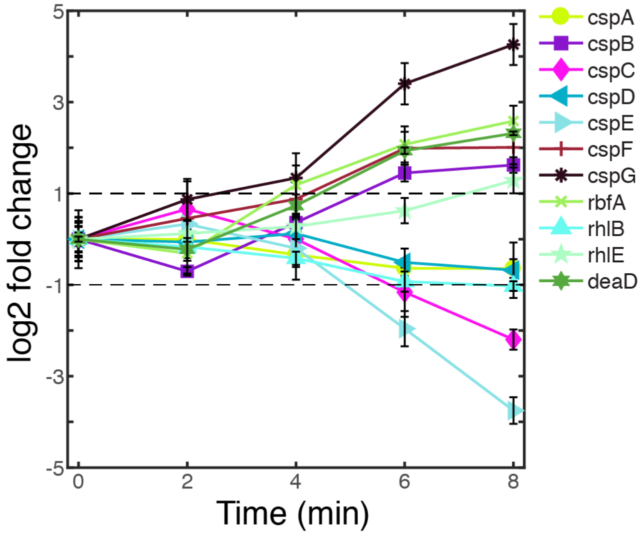

Supplementary Figure 20. RNA-seq log2 fold change of transcript abundance of cold-shock associated genes as a function of time. Cells were induced with 5 ng ml^{-1} aTc. Error bars represent 1 s.d. (n = 2).

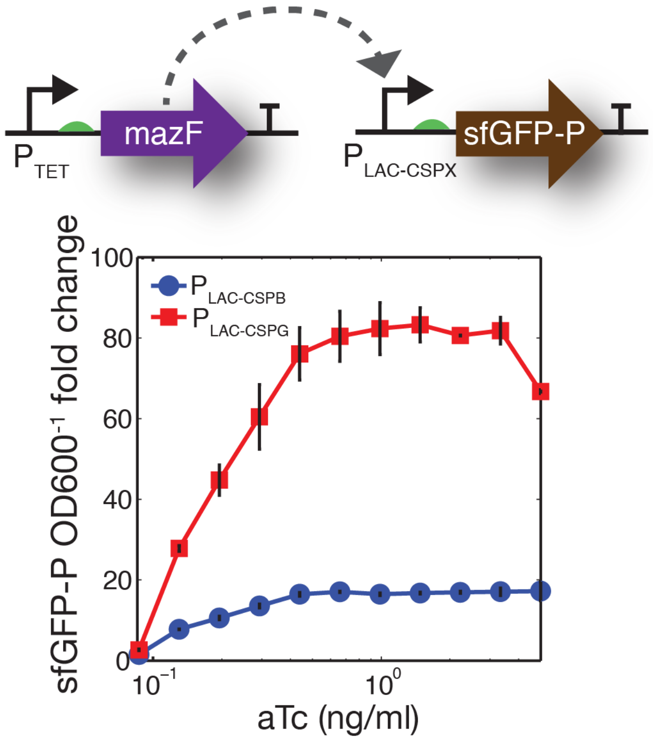

Supplementary Figure 21. Regulatory design elements for building the MazF resource allocator identified by RNA-seq. Tandem promoters composed of P_{LAC} and P_{CspX} (X denotes B or G) driving expression of sfGFP-P were significantly up-regulated in response to MazF activity. MazF was controlled by an aTc-inducible promoter (P_{TET}, top). Fold change of sfGFP-P expression relative to expression prior to induction with MazF as a function of aTc (bottom). Cells were induced with 1 mM IPTG and aTc for 8.2 hr. Error bars represent 1 s.d. (n = 3).

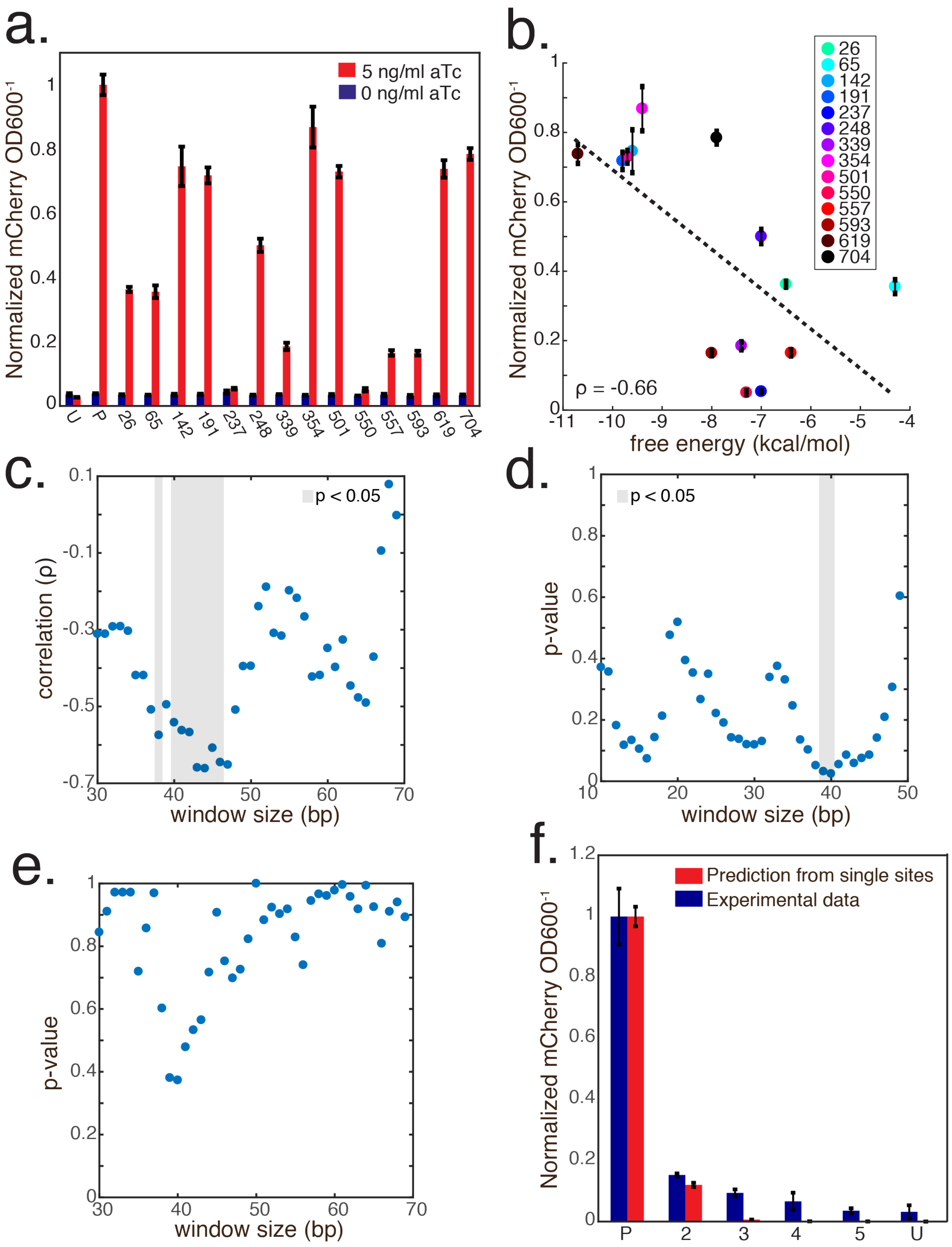

Supplementary Figure 22. The expression levels of 14 *mCherry* sequences containing a single MazF recognition site at different positions were correlated with local secondary structure upstream or across the recognition site. P and U denote mCherry-P or mCherry-U. MazF and

mCherry-X were controlled by an aTc (P_{TET}) and arabinose-inducible promoter (P_{BAD}), respectively. **(a)** *mCherry* variants containing a single MazF recognition site at different positions exhibited a broad range of expression levels in response to MazF. mCherry was normalized by OD600 and the maximum expression across all mCherry variants. Cells were induced with 0.05% arabinose and 0 or 5 ng ml^{-1} aTc for 8 hr. Error bars represent 1 s.d. (n = 2). **(b)** The expression levels of mCherry variants were correlated with the Gibbs free energy (ΔG) of the predicted RNA secondary structure calculated using NUPACK ($\rho = -0.66$, $p = 0.01$ using the Student's t-test). Scatter plot of ΔG (44 nt upstream of the MazF recognition site) vs. mCherry expression. Error bars represent 1 s.d. (n = 2). **(c)** Scatter plot of the correlation coefficient between ΔG vs. mCherry expression across a range of upstream window sizes. Shaded regions denote a statistically significant correlation coefficient ($p < 0.05$ using the Student's t-test). **(d)** Scatter plot of the p-values of the correlation between ΔG and mCherry expression across a range of window sizes spanning upstream and downstream of the MazF recognition site. **(e)** Scatter plot of the p-values of the correlation between ΔG and mCherry expression across a set of window sizes downstream of the MazF recognition site. **(f)** Comparison of experimental measurements and predicted expression levels of *mCherry* sequences containing multiple MazF recognition sites. Cells were induced with 5 ng ml^{-1} aTc and 0.05% arabinose for 8 hr. Fluorescence was divided by OD600 and then normalized by the maximum expression across all conditions. The blue bars denote the predicted expression level of the multi-site *mCherry* sequences by computing the product of the expression levels of *mCherry* sequences containing single sites in (a). MazF recognition sites were located at the following positions: 2 (501, 557); 3 (237, 501, 557); 4 (237, 501, 557, 593); 5 (191, 237, 501, 557, 593). Error bars for the experimental data represent 1 s.d. (n = 3). Uncertainty propagation based on the standard deviations of the single site data in (a) was used to compute the error of the predicted values.

Supplementary Table I
List of strains and plasmids

Strain or plasmid	Description
S1	KTS022IG[1]; mazF::Δ
S2	BW25113; mazF::Δ; SafeSite1::tetR-P_{TET}-mazF
S3	BW25113; mazF::Δ
P1	pBbS2k-P_{TET}-mazF-U
P2	pBbS2k-P_{TET} mazF-P
P4	pBbA8k-P_{BAD}-mCherry-P
P5	pBbA8k-P_{BAD}-mCherry-U
P6	pBbA6c-P_{LAC}-mCherry-P
P7	pBbA6c-P_{LAC}-mCherry-U
P8	pBbA6c-P_{LAC}-gdh-U
P9	pBbA6c-P_{LAC}-gdh-P
P10	pBbA6c-P_{LAC}-sfGFP-P-gdh-U
P11	pBbA6c-P_{LAC}-sfGFP-P-gdh-P
P12	pBbS6c-P_{LAC}-T7-P
P13	pBbS6c-P_{LAC}-T7-U
P14	pVRa-P_{T7}-mCherry-P-gdh-P
P15	pVRa-P_{T7}-mCherry-P-gdh-U
P16	pBbS6c-lacI-W220F[2]-P_{LAC}-rnr-P
P17	pBbS6c-lacI-W220F[2]-P_{LAC}-tsf-P
P18	pBbS6c-lacI-W220F[2]-P_{LAC}-tsf-P; UNS4[3]-$P_{apFAB321}$[4]-rnr-P-UNS6
P19	pVRa-P_{T7}-mCherry-U
P20	pVRa-P_{T7}-mCherry-P
P21	pBbS8k-P_{BAD}-mCherry-U
P22	pBbS8k-P_{BAD}-mCherry-P
P23	pBbS8k-P_{BAD}-mCherry-26
P24	pBbS8k-P_{BAD}-mCherry-65
P25	pBbS8k-P_{BAD}-mCherry-142
P26	pBbS8k-P_{BAD}-mCherry-191
P27	pBbS8k-P_{BAD}-mCherry-237
P28	pBbS8k-P_{BAD}-mCherry-248
P29	pBbS8k-P_{BAD}-mCherry-339
P30	pBbS8k-P_{BAD}-mCherry-354
P31	pBbS8k-P_{BAD}-mCherry-501
P32	pBbS8k-P_{BAD}-mCherry-550
P33	pBbS8k-P_{BAD}-mCherry-557
P34	pBbS8k-P_{BAD}-mCherry-593
P35	pBbS8k-P_{BAD}-mCherry-619
P36	pBbS8k-P_{BAD}-mCherry-704
P37	pBbS2k-P_{TET}-mazF-1
P38	pBbS2k-P_{TET}-mazF-2
P39	pBbS2k-P_{TET}-mazF-3
P40	pBbS2k-P_{TET}-mazF-4
P41	pBbS2k-P_{TET}-mazF-5
P42	pBbS2k-P_{TET}-mazF-10_position_159
P43	pBbS2k-P_{TET}-mazF-11_position_159

P44	pBbA6c-$P_{LAC-CSPB}$-cspB(5'UTR)-(14AA)cspB-sfGFP-P
P45	pBbA6c-$P_{LAC-CSPG}$-cspG(5'UTR)-(14AA)cspG-sfGFP-P

Supplementary Table I. List of strains and plasmids used in this study. S and P denote strains and plasmids, respectively. Constructs were derived from the indicated references.

Supplementary Table II
List of species in MazF resource allocation model

Model species	Description
m_p	p mRNA
m_r	r mRNA
m_f	mazF mRNA
m_{FP}	FP mRNA
m_e	mazE mRNA
p	p protein
r	r protein
FP	FP protein
mazFp	MazF protein
mazFpd	MazF dimer
mazEp	Maze protein
rp	m_p-r complex
rr	m_r-r complex
rmf	m_f-r complex
rFP	m_{FP}-r complex
rme	m_e-r complex
pf	m_p-mazFpd complex
rf	m_r-mazFpd complex
ff	m_f-mazFpd complex
fe	m_e-mazFpd complex
cef	mazFpd-mazEp complex

Supplementary Table III
List of parameters for the MazF resource allocation model

Parameter	Description	Value	Unit	Reference(s)
α_p	m_p transcription rate	1200	nM min^{-1}	[5]
α_r	m_r transcription rate	6	nM min^{-1}	[6]
α_f	m_f transcription rate	0-60	nM min^{-1}	[6]
α_{FP}	m_{FP} transcription rate	0.06	nM min^{-1}	[6]
k_d	mRNA degradation rate	0.252	min^{-1}	[6,7]
k_{deg}	mRNA degradation rate catalyzed by mazFpd	2.52	min^{-1}	[8]
k_f	Forward binding rate	60	nM^{-1} min^{-1}	We assumed the on-rate is diffusion-limited.

k_{rr}	Reverse binding rate of ribosome (r) to m_p, m_f, m_e, m_{FP}	6e4	min^{-1}	[5]
k_{rrR}	Reverse binding rate of ribosome (r) to mRNA	1.2e4	min^{-1}	[5]
k_{trans}	Translation rate	4.002	min^{-1}	[6]
k_{rfp}	m_p-mazFpd reverse binding rate	2.4e3	min^{-1}	[9]
k_{rff}	m_f-mazFpd reverse binding rate	2.39e3-6e5	min^{-1}	[9]
k_{rfr}	m_r-mazFpd reverse binding rate	6e3	min^{-1}	[9]
k_{rd}	mazFp reverse dimerization binding rate	6e3	min^{-1}	Free parameter
k_{pdeg}	Rate of protein degradation	0.0084	min^{-1}	[10]
k_{pdegFP}	Rate of FP protein degradation	0.0017	min^{-1}	[10]
P_{tot}	Total cellular protein concentration	10^6	nM	[11]
α_e	Maximum transcription rate for m_e	0-60	nM min^{-1}	[6]
k_{pdegE}	Rate of MazEp protein degradation	0.0231	min^{-1}	[6]
K_{qe}	Binding affinity (threshold) of m_e transcription rate	6e3	nM	Free parameter
k_{rfe}	mazFpd-m_e reverse binding rate	4440	min^{-1}	[9]
k_{rseq}	mazFpd-mazEp reverse binding rate	6e3	min^{-1}	[9]

Supplementary Table IV
List of qPCR primers and probes

Gene	Forward	Reverse	Probe
rrsA	GTCAGCTCGTGTTGTGA AATG	CCCACCTTCCTCCAGT TTATC	ACGAGCGCAACCCTTATC CTTTGT
cysG	TCTACGACCGTCTGGTTT CT	CGCAGCAGGATCTGG TTAAT	TATGAATCTGGTACGCCG CGATGC
mazF	GGTATGTGTCTGTGTGTT CCTT	CCTTTCTTCGTTGCTC CTCTT	CGTGATGGCTAGCGTTAG CTGAT
mChe rry	CAGGATGGCGAGTTCAT CTATAA	GGATACATGCGTTCG CTAGA	AAAGACGATGGGTTGGGA GGCG

Supplementary Table V
List of genes in RNA-seq clustering analysis

Cell envelope (TIGRFAM) CLUSTER K1	murE(b0085),murF(b0086),mraY(b0087),murD(b0088),murC(b0091),lpxD(b0179),metQ(b0197),mrdA(b0635),pgaD(b1021),lpxL(b1054),rfbC(b2038),rfbA(b2039),mreD(b3249),slp(b3506),bcsG(b3538),rfe(b3784),rffH(b3789),rffM(b3794),murI(b3967),murB(b3972)
Protein synthesis (TIGRFAM) CLUSTER K1	rpsT(b0023),ileS(b0026),rluA(b0058),gluQ(b0144),frr(b0172),tilS(b0188),proS(b0194),cysS(b0526),miaB(b0661),glnS(b0680),rlmC(b0859),infA(b0884),serS(b0893),rpsA(b0911),prfA(b1211),tyrS(b1637),pheT(b1713),pheS(b1714),rpmI(b1717),aspS(b1866),metG(b2114),yeiP(b2171),prmB(b2330),gltX(b2400),hisS(b2514),iscS(b2530),raiA(b2597),trmD(b2607),rpsP(b2609),queD(b2765),lysS(b2890),rpsO(b3165),truB(b3166),hpf(b3203),rpsD(b3296),rplF(b3305),rpsQ(b3311),rplP(b3313),rplV(b3315),tufA(b3339),fusA(b3340),trpS(b3384),glyS(b3559),rpmH(b3703),mnmE(b3706),mnmG(b3741),typA(b3871),trmA(b3965),tufB(b3980),efp(b4147),rpsR(b4202),rplI(b4203),valS(b4258),rimI(b4373)
Translation factors (TIGRFAM) CLUSTER K1	frr(b0172),infA(b0884),prfA(b1211),yeiP(b2171),raiA(b2597),hpf(b3203),tufA(b3339),fusA(b3340),typA(b3871),tufB(b3980),efp(b4147)
tRNA aminoacetylation (TIGRFAM) CLUSTER K1	ileS(b0026),proS(b0194),cysS(b0526),glnS(b0680),serS(b0893),tyrS(b1637),pheT(b1713),pheS(b1714),aspS(b1866),metG(b2114),gltX(b2400),hisS(b2514),lysS(b2890),trpS(b3384),glyS(b3559),valS(b4258)
Energy metabolism - amino acids and amines (TIGRFAM) CLUSTER K2	putA(b1014),gcvP(b2903),gcvH(b2904),gcvT(b2905),ansB(b2957),tdh(b3616),tnaA(b3708),aspA(b4139)
Energy metabolism - anaerobic (TIGRFAM) CLUSTER K2	narG(b1224),narH(b1225),narI(b1227),glpA(b2241),glpB(b2242),glpC(b2243)
Energy metabolism (TIGRFAM) CLUSTER K2	aceF(b0115),acnB(b0118),ykgF(b0307),cyoD(b0429),cyoC(b0430),cyoB(b0431),pgm(b0688),sdhC(b0721),sdhD(b0722),sucB(b0727),sucC(b0728),sucD(b0729),cydB(b0734),ybgT(b4515),pflB(b0903),mgsA(b0963),wrbA(b1004),putA(b1014),narG(b1224),narH(b1225),narI(b1227),acnA(b1276),fdnG(b1474),rsxA(b1627),rsxB(b1628),rsxG(b1631),rsxE(b1632),gloA(b1651),grxD(b1654),eda(b1850),edd(b1851),pykA(b1854),gatZ(b2095),gatY(b2096),ccmF(b2196),napG(b2205),mqo(

	b2210),glpA(b2241),glpB(b2242),glpC(b2243),nuoN(b2276),nuoM(b2277),nuoL(b2278),nuoI(b2281),nuoG(b2283),nuoE(b2285),nuoC(b2286),ackA(b2296),pta(b2297),glk(b2388),trxC(b2582),eno(b2779),sdaB(b2797),gcvP(b2903),gcvH(b2904),gcvT(b2905),rpiA(b2914),fbaA(b2925),tktA(b2935),ansB(b2957),tdcG(b4471),tdcE(b3114),garK(b3124),mdh(b3236),pck(b3403),malP(b3417),glgP(b3428),grxC(b3610),gpmM(b3612),tdh(b3616),tnaA(b3708),atpD(b3732),atpB(b3738),fdoI(b3892),tpiA(b3919),glpK(b3926),fsaB(b3946),aceB(b4014),nrfE(b4074),aspA(b4139),frdA(b4154),queG(b4166),deoC(b4381)
Nitrogen (TIGRFAM) CLUSTER K2	napA(b2206),nirB(b3365)
Protein modification (TIGRFAM) CLUSTER K2	pflA(b0902),prc(b1830),hypA(b2726),aslB(b3800),birA(b3973),nrdG(b4237),yjjW(b4379)
Energy metabolism – TCA* (TIGRFAM) CLUSTER K3	acnB(b0118),sdhC(b0721),sdhD(b0722),sucB(b0727),sucC(b0728),sucD(b0729),acnA(b1276),mqo(b2210),mdh(b3236),aceB(b4014),frdA(b4154)
ArcA (RegulonDB) CLUSTER K2	caiE(b0035),aceE(b0114),aceF(b0115),lpd(b0116),acnB(b0118),fadE(b0221),betT(b0314),cyoD(b0429),cyoC(b0430),cyoB(b0431),dcuC(b0621),sdhC(b0721),sdhD(b0722),sucB(b0727),sucC(b0728),sucD(b0729),cydB(b0734),cydC(b0886),pflB(b0903),ndh(b1109),prfA(b1211),oppB(b1244),oppC(b1245),oppD(b1246),acnA(b1276),tpx(b1324),gatD(b2091),gatC(b2092),gatA(b2094),gatZ(b2095),gatY(b2096),glpA(b2241),glpB(b2242),glpC(b2243),nuoN(b2276),nuoM(b2277),nuoL(b2278),nuoK(b2279),nuoI(b2281),nuoH(b2282),nuoG(b2283),nuoE(b2285),nuoC(b2286),nuoA(b2288),ackA(b2296),grcA(b2579),glcG(b2977),glcF(b4467),hybB(b2995),mdh(b3236),rpsQ(b3311),rplP(b3313),rplV(b3315),rplC(b3320),dctA(b3528),sodA(b3908),aceB(b4014),ubiC(b4039),cadA(b4131),treC(b4239)
ArgR (RegulonDB) CLUSTER K3	artJ(b0860),hisQ(b2308),hisJ(b2309),pnp(b3164),rpsO(b3165),truB(b3166),rbfA(b3167)
CRP (RegulonDB) CLUSTER K2	caiE(b0035),nadC(b0109),pdhR(b0113),aceE(b0114),aceF(b0115),lpd(b0116),acnB(b0118),dksA(b0145),yaeQ(b0190),nlpE(b0192),araJ(b0396),tsx(b0411),cyoD(b0429),cyoC(b0430),cyoB(b0431),hupB(b0440),entC(b0593),entE(b0594),cstA(b0598),nagA(b0677),sdhC(b0721),sdhD(b0722),sucB(b0727),sucC(b0728),sucD(b0729),fiu(b0805),pflB(b0903),aroA(b0908),ompF(b0929),ompA(b0957),ycdZ(b1036),hlyE(b118

	2),sohB(b1272),acnA(b1276),feaB(b1385),uxaB(b1521),marR(b1530),malY(b1622),sodB(b1656),manX(b1817),manY(b1818),manZ(b1819),gatD(b2091),gatC(b2092),gatA(b2094),gatZ(b2095),gatY(b2096),cdd(b2143),preA(b2147),mglC(b2148),mglA(b2149),mglB(b2150),galS(b2151),cirA(b2155),yeiP(b2171),gyrA(b2231),ubiG(b2232),nrdB(b2235),yfaE(b2236),glpT(b2240),glpA(b2241),glpB(b2242),glpC(b2243),dsdX(b2365),nupC(b2393),ptsH(b2415),ptsI(b2416),crr(b2417),guaB(b2508),grcA(b2579),pka(b2584),raiA(b2597),srlA(b2702),srlE(b2703),srlD(b2705),mazF(b2782),mazE(b2783),gcvP(b2903),gcvH(b2904),gcvT(b2905),serA(b2913),fbaA(b2925),galP(b2943),ansB(b2957),nupG(b2964),glcC(b2980),tdcG(b4471),tdcF(b3113),tdcE(b3114),tdcC(b3116),tdcB(b3117),tdcA(b3118),pnp(b3164),rpsO(b3165),truB(b3166),rbfA(b3167),mdh(b3236),dusB(b3260),nirB(b3365),nirC(b3367),cysG(b3368),pck(b3403),glgP(b3428),gntU(b4476),dctA(b3528),yiaM(b3577),mtlD(b3600),ilvN(b3670),ilvB(b3671),tnaC(b3707),tnaA(b3708),bglF(b3722),rbsA(b3749),rbsC(b3750),udp(b3831),glnL(b3869),glnA(b3870),rhaR(b3906),sodA(b3908),glpK(b3926),glpF(b3927),cytR(b3934),aceB(b4014),proP(b4111),dcuA(b4138),aspA(b4139),hflX(b4173),rpsR(b4202),rplI(b4203),treC(b4239),fecE(b4287),fecD(b4288),fecB(b4290),fecA(b4291),yjiY(b4354),osmY(b4376),deoC(b4381),deoB(b4383)
Cra (RegulonDB) CLUSTER K2	pdhR(b0113),aceE(b0114),aceF(b0115),lpd(b0116),acnB(b0118),betT(b0314),cyoD(b0429),cyoC(b0430),cyoB(b0431),cydB(b0734),adhE(b1241),acnA(b1276),marR(b1530),yeaD(b1780),manX(b1817),manY(b1818),manZ(b1819),eda(b1850),edd(b1851),fruB(b2169),glk(b2388),ptsH(b2415),ptsI(b2416),crr(b2417),eno(b2779),fbaA(b2925),glcC(b2980),nirB(b3365),nirC(b3367),cysG(b3368),pck(b3403),mtlD(b3600),gpmM(b3612),envC(b3613),tpiA(b3919),ppc(b3956),aceB(b4014)
DcuR (RegulonDB) CLUSTER K2	dctA(b3528),frdD(b4151),frdC(b4152),frdA(b4154)
FNR (RegulonDB) CLUSTER K2	caiE(b0035),pdhR(b0113),aceE(b0114),aceF(b0115),lpd(b0116),cyoD(b0429),cyoC(b0430),cyoB(b0431),dcuC(b0621),sdhC(b0721),sdhD(b0722),sucB(b0727),sucC(b0728),sucD(b0729),cydB(b0734),moaA(b0781),ompX(b0814),cydC(b0886),dmsA(b0894),dmsC(b0896),pflB(b0903),aspC(b0928),ndh(b1109),hlyE(b1182),prfA(b1211),narL(b1221),narK(b1223),narG(b1224),narH(b1225),narJ(b1226),narI(b1227),adhE(b1241),ompW(b1256),acnA(b1276),tpx(b1324),fdnG(b1474),ynfH(b1590),yecR(b1904),ccmH(b2194),ccmF(b2196),ccmC(b2199),ccmA(b2201),napC(b2202),napB(b2203),napH(b2204),napG(b2205),napA(b2206),napD(b2207),glpT(b2240),glpA(b

	2241),glpB(b2242),glpC(b2243),nuoN(b2276),nuoM(b2277),nuoL(b2278),nuoK(b2279),nuoI(b2281),nuoH(b2282),nuoG(b2283),nuoE(b2285),nuoC(b2286),nuoA(b2288),ackA(b2296),grcA(b2579),trmD(b2607),rpsP(b2609),xdhC(b2868),gcvP(b2903),gcvH(b2904),gcvT(b2905),ansB(b2957),tdcG(b4471),tdcF(b3113),tdcE(b3114),tdcC(b3116),tdcB(b3117),tdcA(b3118),garK(b3124),acrF(b3266),rpsQ(b3311),rplP(b3313),rplV(b3315),rplC(b3320),nirB(b3365),nirC(b3367),cysG(b3368),feoB(b3409),malP(b3417),nikA(b3476),nikB(b3477),nikC(b3478),nikD(b3479),nikE(b3480),pitA(b3493),dppA(b3544),pstA(b3726),pstC(b3727),pstS(b3728),sodA(b3908),katG(b3942),ubiC(b4039),nrfE(b4074),dcuA(b4138),aspA(b4139),frdD(b4151),frdC(b4152),frdA(b4154),nrdG(b4237),nrdD(b4238),arcA(b4401)
Fis (RegulonDB) CLUSTER K2	lpd(b0116),acnB(b0118),thrW(b0244),hupB(b0440),glnX(b0664),valT(b0744),dmsA(b0894),dmsC(b0896),pflB(b0903),ndh(b1109),hlyE(b1182),narK(b1223),narG(b1224),narH(b1225),narJ(b1226),narI(b1227),tpr(b1229),hns(b1237),adhE(b1241),topA(b1274),marR(b1530),mglC(b2148),mglA(b2149),gyrA(b2231),nrdB(b2235),yfaE(b2236),glpT(b2240),glpA(b2241),glpB(b2242),glpC(b2243),nuoN(b2276),nuoM(b2277),nuoL(b2278),nuoK(b2279),nuoI(b2281),nuoH(b2282),nuoG(b2283),nuoE(b2285),nuoC(b2286),nuoA(b2288),gltX(b2400),guaB(b2508),grcA(b2579),mazG(b2781),mazF(b2782),mazE(b2783),fau(b2912),ansB(b2957),glcC(b2980),pnp(b3164),rpsO(b3165),truB(b3166),rbfA(b3167),dusB(b3260),nirB(b3365),nirC(b3367),cysG(b3368),mtlD(b3600),bglF(b3722),gltU(b3757),glnL(b3869),glnA(b3870),trmA(b3965),tufB(b3980),nrfE(b4074),proP(b4111),leuP(b4369),osmY(b4376),deoC(b4381),deoB(b4383)
FlhDC (RegulonDB) CLUSTER K2	gltK(b0653),gltI(b0655),flgC(b1074),flgE(b1076),flgJ(b1081),yecR(b1904),mglC(b2148),mglA(b2149),mglB(b2150),ccmH(b2194),ccmF(b2196),ccmC(b2199),ccmA(b2201),napC(b2202),napB(b2203),napH(b2204),napG(b2205),napA(b2206),napD(b2207),glpA(b2241),glpB(b2242),glpC(b2243),recC(b2822),mdh(b3236),nrfE(b4074)
Fur (RegulonDB) CLUSTER K2	lpd(b0116),fhuA(b0150),fhuD(b0152),fhuB(b0153),cyoD(b0429),cyoC(b0430),cyoB(b0431),fepG(b0589),fepD(b0590),fepB(b0592),entC(b0593),entE(b0594),sdhC(b0721),sdhD(b0722),sucB(b0727),sucC(b0728),sucD(b0729),fiu(b0805),aspC(b0928),ompF(b0929),ndh(b1109),oppB(b1244),oppC(b1245),oppD(b1246),tonB(b1252),mntP(b1821),cirA(b2155),grcA(b2579),nrdF(b2676),exbD(b3005),exbB(b3006),garK(b3124),feoB(b3409),sodA(b3908),metJ(b3938),katG(b3942),fecE(b4287),fecD(b4288),fecB(b4290),fecA(b4291),fecI(b4293),fhuF(b4367),yjjZ(b4567)

GatR (RegulonDB) CLUSTER K2	gatD(b2091),gatC(b2092),gatA(b2094),gatZ(b2095),gatY(b2096)
GcvA (RegulonDB) CLUSTER K2	gcvP(b2903),gcvH(b2904),gcvT(b2905)
GlpR (RegulonDB) CLUSTER K2	glpT(b2240),glpA(b2241),glpB(b2242),glpC(b2243),glpK(b3926),glpF(b3927)
HypT (RegulonDB) CLUSTER K1	metQ(b0197),cydB(b0734),fecE(b4287),fecD(b4288),fecB(b4290),fecA(b4291)
IHF (RegulonDB) CLUSTER K2	sucB(b0727),sucC(b0728),sucD(b0729),glnP(b0810),glnH(b0811),dmsA(b0894),dmsC(b0896),pflB(b0903),ompF(b0929),ndh(b1109),prfA(b1211),narK(b1223),narG(b1224),narH(b1225),narJ(b1226),narI(b1227),pspE(b1308),sodB(b1656),ihfA(b1712),ompC(b2215),atoE(b2223),glpT(b2240),nuoN(b2276),nuoM(b2277),nuoL(b2278),nuoK(b2279),nuoI(b2281),nuoH(b2282),nuoG(b2283),nuoE(b2285),nuoC(b2286),nuoA(b2288),hypA(b2726),glcG(b2977),glcF(b4467),tdcG(b4471),tdcF(b3113),tdcE(b3114),tdcC(b3116),tdcB(b3117),tdcA(b3118),dcuD(b3227),dusB(b3260),nirB(b3365),nirC(b3367),cysG(b3368),uspA(b3495),dppA(b3544),yiaM(b3577),ibpB(b3686),pstA(b3726),pstC(b3727),pstS(b3728),ilvG_1(),ilvG_2(),ilvD(b3771),sodA(b3908),aceB(b4014),ubiC(b4039),nrfE(b4074),adiA(b4117),fimI(b4315),osmY(b4376)
IscR (RegulonDB) CLUSTER K2	napC(b2202),napB(b2203),napH(b2204),napG(b2205),napA(b2206),napD(b2207),iscS(b2530),rnlA(b2630),rnlB(b2631),nrdF(b2676)
LexA (RegulonDB) CLUSTER K1	ftsI(b0084),murE(b0085),murF(b0086),mraY(b0087),murD(b0088),ftsW(b0089),murC(b0091),ftsQ(b0093),ftsA(b0094),uvrB(b0779),ftsK(b0890),yebG(b1848),recD(b2819),dnaG(b3066),lexA(b4043)
MazE-MazF (RegulonDB) CLUSTER K3	mazG(b2781),mazF(b2782),mazE(b2783)
Mlc (RegulonDB) CLUSTER K2	manX(b1817),manY(b1818),manZ(b1819),ptsH(b2415),ptsI(b2416),crr(b2417)
ModE (RegulonDB) CLUSTER K2	moaA(b0781),dmsA(b0894),dmsC(b0896),narL(b1221),oppB(b1244),oppC(b1245),oppD(b1246),ccmH(b2194),ccmF(b2196),ccmC(b2199),ccmA(b2201),napC(b2202),napB(b2203),napH(b2204),napG(b2205),napA(b2206),napD(b2207),deoC(b4381),deoB(b4383)
MraZ (RegulonDB) CLUSTER K1	ftsI(b0084),murE(b0085),murF(b0086),mraY(b0087),murD(b0088),ftsW(b0089),murC(b0091),ftsQ(b0093),ftsA(b0094),mioC(b3742)

NarL (RegulonDB) CLUSTER K2	cydC(b0886),dmsA(b0894),dmsC(b0896),pflB(b0903),narK(b1223),narG(b1224),narH(b1225),narJ(b1226),narI(b1227),adhE(b1241),fdnG(b1474),ynfH(b1590),ccmH(b2194),ccmF(b2196),ccmC(b2199),ccmA(b2201),napC(b2202),napB(b2203),napH(b2204),napG(b2205),napA(b2206),napD(b2207),nuoN(b2276),nuoM(b2277),nuoL(b2278),nuoK(b2279),nuoI(b2281),nuoH(b2282),nuoG(b2283),nuoE(b2285),nuoC(b2286),nuoA(b2288),hybB(b2995),nirB(b3365),nirC(b3367),cysG(b3368),nikA(b3476),nikB(b3477),nikC(b3478),nikD(b3479),nikE(b3480),ubiC(b4039),nrfE(b4074),dcuA(b4138),aspA(b4139),frdD(b4151),frdC(b4152),frdA(b4154)
NarP (RegulonDB) CLUSTER K2	fdnG(b1474),ccmH(b2194),ccmF(b2196),ccmC(b2199),ccmA(b2201),napC(b2202),napB(b2203),napH(b2204),napG(b2205),napA(b2206),napD(b2207),nirB(b3365),nirC(b3367),cysG(b3368),nrfE(b4074)
NikR (RegulonDB) CLUSTER K2	nikA(b3476),nikB(b3477),nikC(b3478),nikD(b3479),nikE(b3480)
RstA (RegulonDB) CLUSTER K2	ompF(b0929),narG(b1224),narH(b1225),narJ(b1226),narI(b1227)
TdcR (RegulonDB) CLUSTER K2	tdcG(b4471),tdcF(b3113),tdcE(b3114),tdcC(b3116),tdcB(b3117),tdcA(b3118)
TorR (RegulonDB) CLUSTER K2	tnaC(b3707),tnaA(b3708)
NtrC (RegulonDB) CLUSTER K3	glnP(b0810),glnH(b0811),cbl(b1987),hisQ(b2308),hisJ(b2309),yhdW(b3268),yhdX(b3269),yhdY(b3270),yhdZ(b3271),glnL(b3869),glnA(b3870)

Supplementary Table V. Functional (TIGRFAM) and regulatory (Regulon DB) enrichments in RNA-seq clustering analysis. *Not statistically significant (p = 0.051).

Supplementary Note 1

<u>MazF resource allocation model</u>
We constructed an ordinary differential equation (ODE) model that interrogated the role of MazF on ribosome competition. The model included mRNA and protein concentrations of all species. Major protein species (Supplementary Table I contains a complete list of model species) included the unprotected cellular proteome (*p*), ribosomes (*r*), MazF monomer (*mazFp*) and dimer (*mazFpd*), MazE (*mazEp*) and a protected gene (*FP*). For simplicity, we assumed that nutrient levels, cellular energy and RNA polymerase concentrations were constant as a function of time. We assumed that MazE was not expressed ($\alpha_e = 0$) for modeling analyses except Supplementary Fig. 16. Supplementary Table II and III contain a description of model species and parameters and Supplementary Fig. 12 shows the molecular interactions in the model. The Supplementary Software contains MATLAB code for simulation of the model.

Parameters were based on previous literature (see Supplementary Table III). Unknown parameters were estimated using physiologically relevant values for *E coli*. We focused on qualitative circuit behaviors, which were robust to small variations in free parameters. The growth rate function was based on a previous model of growth and gene expression. Here, growth rate is defined as $\lambda = k_{trans}P_{tot}^{-1}([rp]+[rr])$. For steady-state analyses, the model was first simulated to steady-state (t = 278 hr) from an initial condition corresponding to 1 nM for all species. Second, the steady-state species concentrations from the first simulation were used as the initial condition for a second simulation. This two-stage simulation mirrored our experimental design, whereby cells were grown for a period of time prior to inducer administration. The equations are:

$$\frac{d[m_p]}{dt} = \alpha_p - k_f[m_p][r] + k_{rr}[\mathrm{rp}] + k_{trans}[\mathrm{rp}] - k_f[m_p][\mathrm{mazFpd}] + k_{rfp}[\mathrm{pf}] - \lambda[m_p] - k_d[m_p],$$

$$\frac{d[m_r]}{dt} = \alpha_r - k_f[m_r][r] + k_{rrR}[\mathrm{rr}] + k_{trans}[\mathrm{rr}] - k_f[m_r][\mathrm{mazFpd}] + k_{rfr}[\mathrm{rf}] - \lambda[m_r] - k_d[m_r],$$

$$\frac{d[m_f]}{dt} = \alpha_f - k_f[m_f][r] + k_{rr}[\mathrm{rmf}] + k_{trans}[\mathrm{rmf}] - k_f[m_f][\mathrm{mazFpd}] + k_{rff}[\mathrm{ff}] - \lambda[m_f] - k_d[m_f],$$

$$\frac{d[\mathrm{m_{FP}}]}{dt} = \alpha_{FP} - k_f[m_{FP}][r] + k_{rr}[\mathrm{rFP}] + k_{trans}[\mathrm{rFP}] - \lambda[m_{FP}] - k_d[m_{FP}],$$

$$\frac{d[m_e]}{dt} = \alpha_e\left(\frac{[\mathrm{mazFpd}]}{[\mathrm{mazFpd}] + K_{qe}}\right) - k_f[m_e][r] + k_{rr}[\mathrm{rme}] + k_{trans}[\mathrm{rme}] - k_f[m_e][\mathrm{mazFpd}]$$
$$+ k_{rfe}[\mathrm{fe}] - \lambda[m_e] - k_d[m_e],$$

$$\frac{d[p]}{dt} = k_{trans}[\mathrm{rp}] - \lambda[p] - k_{pdeg}[p],$$

$$\frac{d[r]}{dt} = -k_f[m_e][r] + k_{rr}[\mathrm{rme}] + k_{trans}[\mathrm{rme}] - k_f[m_p][r] + k_{rr}[\mathrm{rp}] + k_{trans}[\mathrm{rp}] - k_f[m_r][r] + k_{rrR}[\mathrm{r}$$ [illegible]
$$+ 2k_{trans}[\mathrm{rr}] - k_f[m_f][r] + k_{rr}[\mathrm{rmf}] + k_{trans}[\mathrm{rmf}] - k_f[m_{FP}][r] + k_{rr}[\mathrm{rFP}] + k_{trans}[\mathrm{rFP}]$$
$$- k_{pdeg}[r] - \lambda[r],$$

$$\frac{d[FP]}{dt} = k_{trans}[\mathrm{rFP}] - \lambda[\mathrm{FP}] - k_{pdegFP}[\mathrm{FP}],$$

$$\frac{d[\mathrm{mazFp}]}{dt} = k_{trans}[\mathrm{rmf}] - 2k_f[\mathrm{mazFp}]^2 + 2k_{rd}[\mathrm{mazFpd}] - \lambda[\mathrm{mazFp}] - k_{pdeg}[\mathrm{mazFp}],$$

$$\frac{d[\mathrm{mazFpd}]}{dt} = k_f[\mathrm{mazFp}]^2 - k_{rd}[\mathrm{mazFpd}] - \lambda[\mathrm{mazFpd}] - k_f[m_p][\mathrm{mazFpd}] + k_{rfp}[\mathrm{pf}] + k_{deg}[\mathrm{pf}]$$
$$- k_f[m_r][\mathrm{mazFpd}] + k_{rfr}[\mathrm{rf}] + k_{deg}[\mathrm{rf}] - k_f[m_f][\mathrm{mazFpd}] + k_{rff}[\mathrm{ff}] + k_{deg}[\mathrm{ff}]$$
$$- k_{pdeg}[\mathrm{mazFpd}] - k_f[\mathrm{maze}][\mathrm{mazFpd}] + k_{rseq}[\mathrm{cef}] + k_{rfe}[\mathrm{fe}] + k_{deg}[\mathrm{fe}]$$
$$- k_f[m_e][\mathrm{mazFpd}],$$

$$\frac{d[\mathrm{mazEp}]}{dt} = k_{trans}[\mathrm{rme}] - k_{pdegE}[\mathrm{mazEp}] - \lambda[\mathrm{mazEp}] - k_f[\mathrm{mazEp}][\mathrm{mazFpd}] + k_{rseq}[\mathrm{cef}],$$

$$\frac{d[\mathrm{rp}]}{dt} = k_f[m_p][r] - k_{rr}[\mathrm{rp}] - k_{trans}[\mathrm{rp}] - \lambda[\mathrm{rp}] - k_{pdeg}[\mathrm{rp}],$$

$$\frac{d[rr]}{dt} = k_f[m_r][r] - k_{rrR}[\mathrm{rr}] - k_{trans}[\mathrm{rr}] - \lambda[\mathrm{rr}] - k_{pdeg}[\mathrm{rr}],$$

$$\frac{d[\mathrm{rmf}]}{dt} = k_f[m_f][r] - k_{rr}[\mathrm{rmf}] - k_{trans}[\mathrm{rmf}] - \lambda[\mathrm{rmf}] - k_{pdeg}[\mathrm{rmf}],$$

$$\frac{d[\mathrm{rFP}]}{dt} = k_f[m_{FP}][r] - k_{rr}[\mathrm{rFP}] - k_{trans}[\mathrm{rFP}] - \lambda[\mathrm{rFP}] - k_{pdeg}[\mathrm{rFP}],$$

$$\frac{d[\mathrm{rme}]}{dt} = k_f[m_e][r] - k_{rr}[\mathrm{rme}] - k_{trans}[\mathrm{rme}] - \lambda[\mathrm{rme}] - k_{pdeg}[\mathrm{rme}],$$

$$\frac{d[\mathrm{pf}]}{dt} = k_f[m_p][\mathrm{mazFpd}] - k_{rfp}[\mathrm{pf}] - \lambda[\mathrm{pf}] - k_{pdeg}[\mathrm{pf}] - k_{deg}[\mathrm{pf}],$$

$$\frac{d[\mathrm{rf}]}{dt} = k_f[m_r][\mathrm{mazFpd}] - k_{rfr}[\mathrm{rf}] - \lambda[\mathrm{rf}] - k_{pdeg}[\mathrm{rf}] - k_{deg}[\mathrm{rf}],$$

$$\frac{d[\mathrm{ff}]}{dt} = k_f[m_f][\mathrm{mazFpd}] - k_{rff}[\mathrm{ff}] - \lambda[\mathrm{ff}] - k_{pdeg}[\mathrm{ff}] - k_{deg}[\mathrm{ff}],$$

$$\frac{d[\text{fe}]}{dt} = k_f[m_e][\text{mazFpd}] - k_{rfe}[\text{fe}] - \lambda[\text{fe}] - k_{pdeg}[\text{fe}] - k_{deg}[\text{fe}],$$
$$\frac{d[\text{cef}]}{dt} = k_f[\text{mazEp}][\text{mazFpd}] - k_{rseq}[\text{cef}] - \lambda[\text{cef}] - k_{pdeg}[\text{cef}].$$

REFERENCES

1. Solomon, K. V., Moon, T. S., Ma, B., Sanders, T. M. & Prather, K. L. J. Tuning primary metabolism for heterologous pathway productivity. *ACS Synth. Biol.* **2,** 126–135 (2013).
2. Gatti-Lafranconi, P., Dijkman, W. P., Devenish, S. R. a & Hollfelder, F. A single mutation in the core domain of the lac repressor reduces leakiness. *Microb. Cell Fact.* **12,** 67 (2013).
3. Lou, C., Stanton, B., Chen, Y.-J., Munsky, B. & Voigt, C. a. Ribozyme-based insulator parts buffer synthetic circuits from genetic context. *Nat. Biotechnol.* **30,** 1137–42 (2012).
4. Mutalik, V. K. *et al.* Precise and reliable gene expression via standard transcription and translation initiation elements. *Nat. Methods* **10,** 354–60 (2013).
5. Weiße, A. Y., Oyarzún, D. a., Danos, V. & Swain, P. S. Mechanistic links between cellular trade-offs, gene expression, and growth. *Proc. Natl. Acad. Sci.* **112,** E1038–E1047 (2015).
6. Bremer, H. & Dennis, P. Modulation of chemical composition and other parameters of the cell by growth rate. In Escherichia coli and Salmonella typhimurium. *EcoSal Plus* **3,** (2008).
7. Bernstein, J. A., Khodursky, A. B., Lin, P.-H., Lin-Chao, S. & Cohen, S. N. Global analysis of mRNA decay and abundance in Escherichia coli at single-gene resolution using two-color fluorescent DNA microarrays. *Proc. Natl. Acad. Sci. U. S. A.* **99,** 9697–702 (2002).
8. Garamella, J., Marshall, R., Rustad, M. & Noireaux, V. The All E. coli TX-TL Toolbox 2.0: A Platform for Cell-Free Synthetic Biology. *ACS Synth. Biol.* **5,** 344–355 (2016).
9. Li, G. *et al.* Characterization of Dual Substrate Binding Sites in the Homodimeric Structure of Escherichia coli mRNA Interferase MazF. *J. Mol. Biol.* **357,** 139–150 (2006).
10. Kolodrubetz, D. & Schleif, R. Identification of araC protein on two-dimensional gels, its in vivo instability and normal level. *J. Mol. Biol.* **149,** 133–139 (1981).
11. Milo, R. What is the total number of protein molecules per cell volume? A call to rethink some published values. *BioEssays* **35,** 1050–1055 (2013).
